# Matrix Isolation Spectroscopic and Relativistic Quantum Chemical Study of Molecular Platinum Fluorides PtF_*n*_ (*n*=1–6) Reveals Magnetic Bistability of PtF_4_

**DOI:** 10.1002/chem.202102055

**Published:** 2021-08-19

**Authors:** Gene Senges, Lin Li, Artur Wodyński, Helmut Beckers, Robert Müller, Martin Kaupp, Sebastian Riedel

**Affiliations:** ^1^ Freie Universität Berlin Institut für Chemie und Biochemie–Anorganische Chemie Fabeckstrasse 34/36 14195 Berlin Germany; ^2^ Technische Universität Berlin Institut für Chemie Theoretische Chemie/Quantenchemie Sekr. C7 Strasse des 17. Juni 135 10623 Berlin Germany

**Keywords:** IR spectroscopy, matrix isolation, platinum fluorides, quantum chemistry

## Abstract

Molecular platinum fluorides PtF_*n*_, *n*=1–6, are prepared by two different routes, photo‐initiated fluorine elimination from PtF_6_ embedded in solid noble‐gas matrices, and the reaction of elemental fluorine with laser‐ablated platinum atoms. IR spectra of the reaction products isolated in rare‐gas matrices under cryogenic conditions provide, for the first time, experimental vibrational frequencies of molecular PtF_3_, PtF_4_ and PtF_5_. Photolysis of PtF_6_ enabled a highly efficient and almost quantitative formation of molecular PtF_4_, whereas both PtF_5_ and PtF_3_ were formed simultaneously by subsequent UV irradiation of PtF_4_. The vibrational spectra of these molecular platinum fluorides were assigned with the help of one‐ and two‐component quasirelativistic DFT computation to account for scalar relativistic and spin–orbit coupling effects. Competing Jahn‐Teller and spin–orbit coupling effects result in a magnetic bistability of PtF_4_, for which a spin‐triplet (^3^B_2g_, *D*_2h_) coexists with an electronic singlet state (^1^A_1g_, *D*_4h_) in solid neon matrices.

## Graphical abstract

**Spin cycle**: The IR spectra of molecular platinum fluorides PtF_*n*_ (*n*=1–6) isolated in rare‐gas matrices are presented. PtF_4_ is formed almost quantitatively under blue light radiation of PtF_6_, while PtF_5_ and PtF_3_ were produced by subsequent UV radiation of PtF_4_. The coexistence of a singlet and a triplet electronic spin state of PtF_4_ caused by competing Jahn‐Teller and spin–orbit coupling effects has been verified spectroscopically in the low‐temperature matrix.

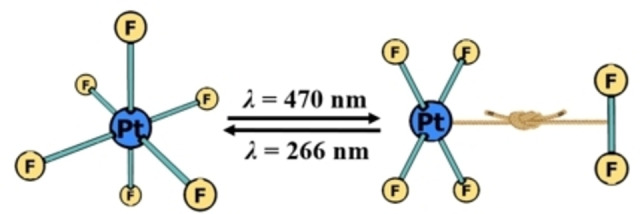

## Introduction

The most common oxidation states for platinum are +2 and +4, but PtF_6_ and the ions PtF_6_^−^ and PtF_6_^2−^ are probably the best known and most investigated binary platinum fluoride species.^[1]^ Unlike the solid mixed‐valence trifluoride (Pt^II^Pt^IV^F_6_),^[2]^ and solid tetrafluoride,^[3]^ platinum pentafluoride is a low‐melting (m.p. 80 °C), deep‐red solid that disproportionates readily to yield PtF_4_ and PtF_6_ upon heating.[^3c^, ^4^] The deep‐red platinum hexafluoride combines high volatility (m.p. 61.3 °C)^[5]^ with the highest electron affinity (EA=7.09 eV)^[6]^ of the known metal hexafluorides. Thus, PtF_6_ is a useful and extremely strong one‐electron oxidizer that has found its place in chemical history as the first species that enabled the synthesis of a stable dioxygenyl salt, [O_2_]^+^ [PtF_6_]^−^, and of the first xenon compounds, likely [FXe]^+^ [PtF_6_]^−^ and [FXe]^+^ [Pt_2_F_11_]^−^.[^1a^, ^1b^, ^7^]

There are numerous investigations on molecular PtF_6_ (see ref. [1b] and references therein), but only very few spectroscopic investigations on molecular fluorides of platinum in lower oxidation states. Platinum monofluoride (PtF) has been studied by microwave,^[8]^ laser absorption,^[9]^ and laser‐induced fluorescence spectroscopy.^[10]^ Very recently we have reported the infrared stretching frequencies of PtF and PtF_2_ embedded in solid neon and argon matrices.^[11]^ PtF_*n*_ (*n*=2–4) were studied by means of high‐temperature Knudsen cell mass spectroscopy and their thermochemistry has been explored.^[12]^ However, experimental spectroscopic investigations on the molecular platinum fluorides PtF_3_, PtF_4_ and PtF_5_ are not yet available.

In this work, we present vibrational spectra and UV transitions of molecular PtF_4_ embedded in cryogenic solid neon and argon matrices. We also report on a first systematic investigation of the molecular platinum fluorides PtF_*n*_ (*n*=1–6) formed independently by two different routes, the reaction of laser‐ablated platinum atoms with elemental fluorine and the selective photodecomposition of PtF_6_. We were also interested in the IR spectroscopic detection of non‐classical PtF_*n*_⋅F_2_ (*n*=4, 5) complexes. The existence of such complexes has so far only been predicted computationally, for example for AuF_5_⋅F_2_ (AuF_7_),^[13]^ and [PtF_5_⋅F_2_] ^−^ (PtF_7_^−^),^[6]^ but has never been verified experimentally. One possible access to such non‐classical difluorine complexes of PtF_4_ could be the photochemically initiated elimination of F_2_ from PtF_6_ in a solid noble‐gas matrix. Our experimental results are supplemented by ab initio CCSD(T) calculations, one‐component quasirelativistic DFT computations^[14]^ that include (spin‐free) scalar relativistic (SR) effects, as well as two‐component quasirelativistic DFT computations^[15]^ including spin–orbit coupling (SOC) effects (for experimental and computational details see the Supporting Information).

In a previous computational study, only even‐numbered molecular platinum fluorides PtF_2*n*_ (*n*=1–4) were studied by scalar relativistic density functional and coupled‐cluster methods.^[16]^ However, more recent studies on PtF_6_ ^[17]^, PtF_6_^2−^,^[18]^ PtX_4_^2−^ (X=F, Cl, Br)^[19]^ and the related PdF_4_ ^[20]^ have shown that SOC effects have a dramatic impact on their electronic structure and spectra. SOC effects can open electronic decay channels, which were closed in non‐relativistic calculations.

**Figure 1:**
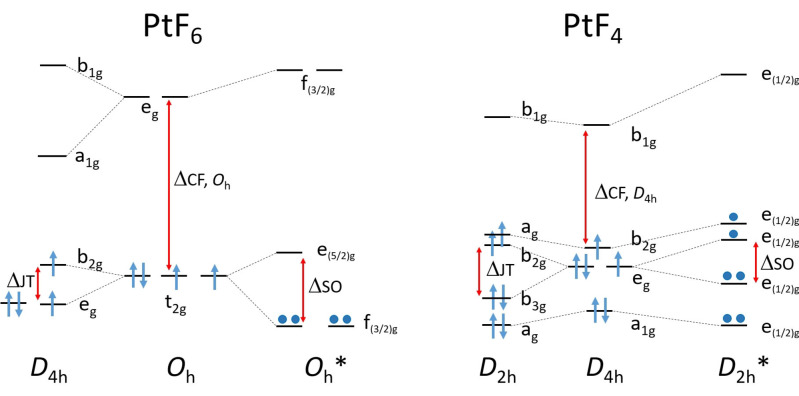
Simplified scheme of Jahn‐Teller (JT) distortion and spin–orbit coupling (SOC) on the platinum 5d orbital splitting of singlet octahedral PtF_6_ (*O*_h_, ^1^A_1g_, left) and triplet square planar PtF_4_ (*D*_4h_, ^3^B_2g_, right). ΔJT and ΔSO indicate the Jahn‐Teller and spin–orbit splitting of degenerate orbitals in the high‐symmetry configuration, respectively, and ΔCF that due to crystal field splitting.

For the neutral PtF_6_ and PtF_4_ molecules, which are most relevant in this work, ligand field theory predicts a triplet ^3^T_1g_ ground state for the t_2g_^4^e_g_^0^ configuration of octahedral PtF_6_ (*O*_h_) and a ^3^E_g_ ground state associated with the a_g_^2^e_g_^3^b_2g_^1^ occupancy of a square planar PtF_4_ (*D*_4h_)^[16]^ (see Figure 1, and ref. [21] for the d‐orbital splitting in octahedral and square planar transition metal complexes). The singly occupied molecular orbitals (MOs) in both molecules are predominantly Pt(5d) orbitals with some π* antibonding Pt−F character. The Jahn‐Teller (JT) theorem predicts a geometrical distortion from the high‐symmetry configuration for degenerate electronic states of such nonlinear molecules, that lowers the symmetry and lifts the degeneracy.^[22]^ Hence, nonrelativistic and scalar‐relativistic electronic structure computations for PtF_6_ predict JT‐distorted *D*_4h_ or *D*_3d_ molecular structures,[^6^, ^16^] but relativistic computations including SOC^[17]^ give, in accordance with experimental structural and spectroscopic data, a diamagnetic octahedral molecule with a closed‐shell singlet ground state (Figure 1).^[1b]^ Thus, PtF_6_ represents the rare case where relativistic spin–orbit splitting leads to a qualitative change of molecular and electronic structure in a stable molecule.^[1b]^

## Results and Discussion

### Conversion of PtF_6_ to PtF_4_

**Figure 2:**
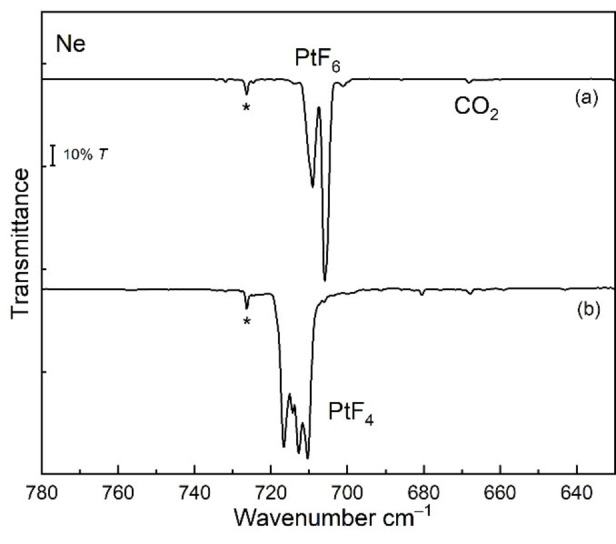
Pt−F stretching band region of Ne matrix‐isolation IR spectra of PtF_6_ and molecular PtF_4_: a) precursor PtF_6_ seeded in excess Ne and co‐deposited for 85 min at 6 K, and b) after irradiation at *λ*=470 nm (LED) for 70 min. Impurity bands are marked by an asterisk.

We have studied the photochemistry of PtF_6_ isolated in solid noble‐gas matrices. PtF_6_ was prepared according to the original protocol of Weinstock et al.,^[5a]^ and its vapor was co‐deposited onto the matrix support together with an excess of pure neon and argon, respectively. In solid neon, the two IR active fundamentals of octahedral PtF_6_ are observed at *ν*_3_=705.6 cm^−1^ (Figure 2) and *ν*_4_=274.6 cm^−1^ (Figure S2.3 in the Supporting Information), where the former is accompanied by a weaker matrix site at 709.1 cm^−1^ (Figure 2). These frequencies are close to previously reported values for PtF_6_ in a solid Ar matrix (*ν*_3_=705.2 cm^−1^, *ν*_4_=274.2 cm^−1^),^[23]^ and reported vapor‐phase frequencies (705, 273 cm^−1^).^[24]^

**Figure 3:**
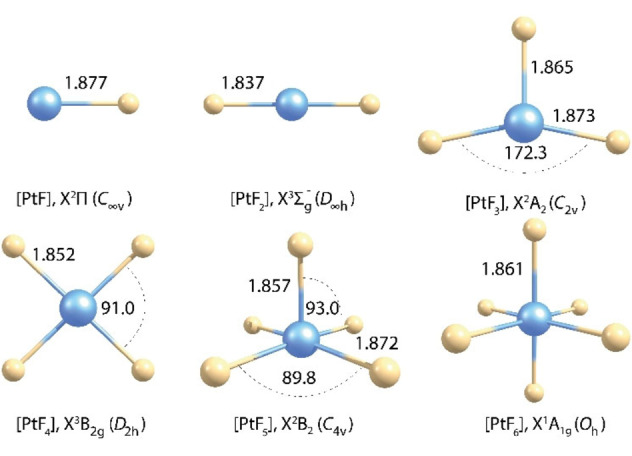
Optimized structures of molecular platinum fluorides PtF_*n*_, *n*=1–6, obtained at the 2c‐X2C‐TPSSh/x2c‐TZVPall‐2c level. Bond lengths are given in Å and angles in degrees.

Upon irradiation of this deposit with blue LED light (*λ*=470 nm) the red color of matrix‐isolated PtF_6_ diminished and the deposit became almost colorless (Figure S2.1). In the Pt−F stretching region four closely spaced new IR bands appeared at wavenumbers between 710 and 717 cm^−1^ (Figure 2). A similar band pattern occurred in all subsequent photolysis experiments, which indicates that these bands are likely associated with a single new species. It is assumed that the photodecomposition of PtF_6_ either cleaves a single Pt−F bond to give a fluorine free radical and PtF_5_, or it leads to the elimination of F_2_ and molecular PtF_4_. For square pyramidal PtF_5_ (Figure 3) at least two different Pt−F stretching bands, a strong equatorial asymmetric PtF_4_ stretching mode and a much weaker axial Pt−F’ stretch, can be expected, while a square planar PtF_4_ species will show only a single IR‐active Pt−F stretching band. As mentioned above, the predicted ^3^E_g_ ground state for a square planar PtF_4_ (*D*_4h_; Figure 1) will be subject to a Jahn‐Teller (JT) distortion,^[16]^ that lowers the symmetry and lifts the degeneracy.^[22]^ JT distortions of tetragonal *D*_4h_ molecules have been studied much less frequently than those of trigonal or octahedral molecules.^[25]^ Generally, for centrosymmetric molecules JT effects will preserve the inversion symmetry, and the non‐degenerate vibrational modes b_1g_ and b_2g_ are JT active in *D*_4h_ molecules.^[22]^ As a consequence, JT distortion of square planar PtF_4_ will lead to a splitting of the single degenerate Pt−F mode and therefore two closely spaced vibrational Pt−F bands of almost equal intensity are expected to appear in the IR spectrum. Such a two‐band spectrum is indeed shown in Figure 2 for the observed photo‐dissociation product of PtF_6_, where both bands reveal an additional matrix‐site splitting like the PtF_6_ precursor band. This spectrum already provides strong evidence for the formation of JT distorted planar PtF_4_ (*D*_2h_ symmetry). After prolonged irradiation, the photo‐initiated transformation of PtF_6_ to PtF_4_ is almost quantitative and surprisingly selective, since no other bands occurred in the Pt−F stretching region. This allowed us to additionally measure several much weaker bands of PtF_4_, such as two combination bands at 1281.8 and 1393.5 cm^−1^ (Figure S2.2), as well as three weak bands in the far‐IR region at 270.5, 248.1, and 221.1 cm^−1^ (Table 1, Figure S2.3). The combination band positions in the infrared spectrum provide useful estimates for the two infrared‐inactive Pt−F stretching modes of PtF_4_ (a_g_+b_1g_). For example, the corresponding two combination bands observed for PtF_6_ in solid Ne at 1305.8 cm^−1^ (*ν*_2_+*ν*_3_) and 1361.0 cm^−1^ (*ν*_1_+*ν*_3_; Figure S2.2), provide upper limits for the Raman bands of *ν*_2_=600 cm^−1^ (e_g_) and *ν*_1_=655 cm^−1^ (a_1g_), which are close to the reported Raman bands of PtF_6_ at 601 and 655 cm^−1^, respectively.^[24]^ Accordingly, from the two combination bands of PtF_4_, we estimate upper limits for two unobserved Raman bands of 677 cm^−1^ (*ν*_3_) and 569 cm^−1^, in good agreement with two‐component DFT calculations (*ν*_4_, Table 1).

**Table 1:** Observed and computed vibrational frequencies [cm^−1^] of PtF_4_ (*D*_4h_).

*ν*_i_, i=1–9^[a]^	Exp.^[b]^	Calcd. (Int.)^[c]^				Modes^[d]^
		^3^B_2g_ (*D*_2h_)		^1^A_g_ (*D*_4h_)		
		1c‐X2C	2c‐X2C	1c‐X2C	2c‐X2C	
*ν*_1_ (B_3u_)	716.6 (714.4)	712 (93)	707 (97)	705 (100)	707 (100)	*ν*_as_(PtF), antisym. stretch (x)
*ν*_2_ (B_2u_)	712.7 (710.5)	701 (100)	702 (100)	705 (100)	706 (100)	*ν*_as_(PtF), antisym. stretch (y)
*ν*_3_ (A_g_)	677^[e]^	685 (0)	675 (0)	692 (0)	681 (0)	*ν*_s_(PtF), in phase stretch
*ν*_4_ (B_1g_)	*569* ^[e]^	426 (0)	579 (0)	629 (0)	599 (0)	*ν*(PtF), out of phase stretch
*ν*_5_ (A_g_)	n. o.	272 (0)	255 (0)	305 (0)	260 (0)	δ_s_(FPtF), in plane scissor
*ν*_6_ (B_3u_)	270.5	256 (5)	252 (4)	215 (12)	240 (6)	δ_as_(FPtF), antisym. bend (x)
*ν*_8_ (B_2u_)	248.1 (247.1)	223 (8)	223 (7)	216 (12)	239 (6)	δ_as_(FPtF), antisym. bend (y)
*ν*_7_ (B_1u_)	221.1	234 (6)	238 (5)	311 (6)	233 (6)	δ_oop_, out of plane bend (z)
*ν*_9_ (A_u_)	n. o.	165(0)	161 (0)	233 (0)	176 (0)	δ_oop_, out of plane pucker

[a] In parentheses: symmetry species for the *D*_2h_ symmetry. [b] Ne matrix, 6 K, matrix‐site bands are given in parentheses, n.o.=not observed. [c] Values calculated at the 1c‐ and 2c‐X2C‐TPSSh levels; relative intensities are given in parentheses. [d] Tentative mode description. [e] Estimated from the combination bands *ν*_2_+*ν*_4_=1281.8 cm^−1^ and *ν*_1_+*ν*_3_=1393.5 cm^−1^ (^3^B_2g_ state) neglecting anharmonicity (Figure S2.2).

Investigating the photo‐decomposition of PtF_6_ in a solid argon matrix we found a different photo‐behavior than in solid neon. In solid argon the PtF_6_ band is also depleted by green light LED radiation of *λ*=528 nm (Figure S2.4). This photo‐decomposition is much less efficient than the blue light charge‐transfer (CT) excitation (Figure 2), and a new band occurred at 679.8 cm^−1^ (Figure S2.4). The new feature is assigned to the degenerate e‐type Pt−F stretching mode of molecular PtF_5_ based on the observed band positions and its photo‐behavior in further experiments (see below and Tables 2 and S5.1). For the lowest‐energy electronic state of PtF_5_ (^2^B_2_, *C*_4v_), a slightly distorted square pyramidal structure with an axial and four longer equatorial Pt−F bonds is predicted (Figure 3). The much weaker computed axial Pt−F stretching vibration of PtF_5_ (Table S5.2) was not detected experimentally. Under subsequent blue light radiation (*λ*=455 nm) PtF_4_ was formed again and the bands due to PtF_6_ and PtF_5_ diminished simultaneously (Figure S2.4). The frequency shift between the two Pt−F stretching bands of PtF_4_ isolated in solid argon (Table 2) is smaller than that in neon, which likely indicates a smaller distortion of the PtF_4_ molecule from the high‐symmetry *D*_4h_ structure in the argon matrix.

**Table 2:** Observed and computed IR active Pt−F stretching frequencies [cm^−1^] for PtF_*n*_ (*n*=1–6).

	Sym.	State	Exp.^[a]^		Calcd.^[b]^		Modes^[c]^
			Ne	Ar	1c‐X2C	2c‐X2c	
PtF	*C*_∞v_	^2^Σ^+^	605.6	590.0	618 (100)	620 (100)	ν (^195^Pt−F), Σ^+^
PtF_2_	*D*_∞h_	^3^Σ_g_^−^	710.1 (706.1)	695.6	735 (100)	710 (100)	ν_as_ (^195^Pt−F_2_), Σ_u_^+^
PtF_3_	*C*_2v_	^2^A_2_	(685.7) 682.4 (680.5)	(671.5) 669.4	690 (100)	684 (100)	ν_as_ (^195^Pt−F_2_), B_1_
			635.3	–	645 (16)	624 (23)	ν (^195^Pt−F′), A_1_
PtF_4_	*D*_2h_	^3^B_2g_	716.6 (714.4)	709.5	712 (93)	707 (97)	ν (^195^Pt−F_2_), B_3u_
			712.7 (710.5)	708.1	701 (100)	702 (100)	ν (^195^Pt−F_2_′), B_2u_
PtF_5_	*C*_4v_	^2^B_2_	(694.8) 691.2	679.8	685 (100)	684 (100)	ν_as_ (^195^Pt−F_4_), E
			–	–	697 (4) ^[d]^	646 (9) ^[d]^	ν (^195^Pt−F′), A_1_
PtF_6_	*O*_h_	^1^A_1g_	(709.1) 705.6	705.5 (701.8)		698 (100)	ν_as_ (^195^PtF_6_), T_1u_
			274.6			276 (8)	δ_as_(^195^PtF_6_), T_1u_

[a] Matrix sites are given in parentheses. [b] Values calculated at one and two‐component X2C‐TPSSh level, respectively. Relative IR intensities are given in parentheses and a complete list of computed frequencies is provided in the Supporting Information. [c] Tentative mode description; Platinum isotope splitting is experimentally not resolved. Computed isotope splitting is listed in the Supporting Information. [d] Computed frequencies and relative intensities (in parentheses) of ν_s_ (^195^Pt−F_4_), A_1_, are at 665 cm^−1^ (0, 1c‐X2C) and 670 cm^−1^ (3, 2c‐X2C), respectively.

**Figure 4:**
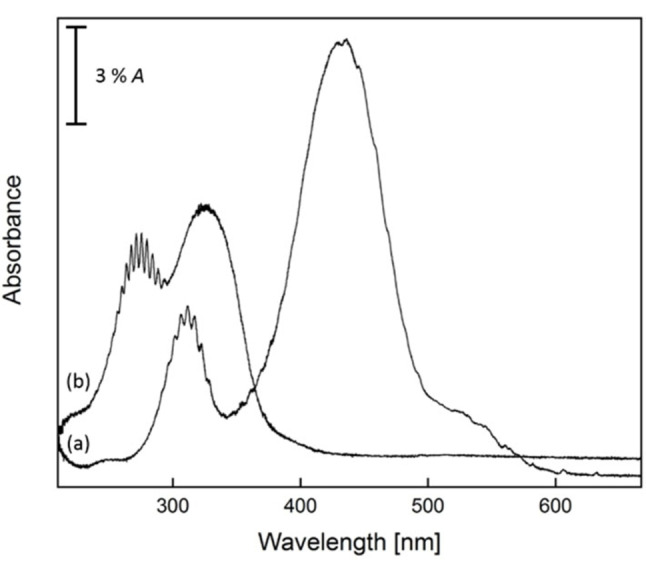
UV/Vis spectra (resolution: 0.1 nm) of a) PtF_6_ and b) PtF_4_ in the range from 667 to 210 nm. Spectrum (a) was recorded from PtF_6_ isolated in solid neon after deposition at 6 K for 36 min and spectrum (b) was obtained after irradiation of the initial deposit with blue LED light (*λ*=470 nm) for 75 min.

UV/Vis spectra of the PtF_6_ precursor embedded in a neon matrix were recorded before and after the *λ*=470 nm irradiation (Figures 4, S2.5, and S2.6). In agreement with reported gas‐phase spectra^[26]^ and with spectra obtained from PtF_6_ in a solid nitrogen matrix[^17b^, ^23^] two strong F‐to‐Pt charge‐transfer (CT) bands of PtF_6_ were observed centered at 436 and 312 nm (Figures 4a and S2.5). Both bands reveal a resolved vibrational structure with an averaged vibrational spacing of 498±10 and 551±12 cm^−1^, respectively (Table S3.1). These frequencies were previously assigned to the totally symmetric stretching modes in the corresponding excited states.[^23^, ^26^] The UV spectrum obtained for PtF_4_ is very similar to that of PtF_6_ (Figure 4b). There are two strong CT bands blue shifted from the PtF_6_ bands with *λ*_max_=325 nm and 272 nm. The lower‐wavelength band revealed a well‐resolved vibrational progression with an average spacing of 542±15 cm^−1^ (Figure S2.6, Table S4.1).

### Quantum‐chemical calculations on PtF_6_ and PtF_4_

To support our assignments, quasi‐relativistic DFT (density functional theory) calculations were carried out on the molecular platinum fluorides PtF_*n*_ (*n*=1–6) at scalar relativistic one‐component (1c‐X2C)^[14]^ and two‐component (2c‐X2C and 2c‐ZORA)[^15^, ^27^] levels to include spin–orbit coupling (SOC) effects (for computational details see the Supporting Information). Our computed structures and vibrational frequencies at the 2c‐X2C level for the PtF_6_ ground state (*O*_h_ symmetry) are fully consistent with previous results (Table S3.2).^[17]^ We note that SOC splits the degenerate spin‐free t_2g_ MOs into a fourfold degenerate HOMO ((f_(3/2)g_)^4^ configuration) and an unoccupied twofold degenerate LUMO (e_(5/2)g_) level (Figure 1). Based on 2c‐X2C and 2c‐ZORA TDDFT calculations the observed two strong CT bands of PtF_6_ (Figures 4 and S2.6) are assigned to threefold degenerate (T_1u_) excitations involving transitions from occupied fluorine lone‐pair (π(F)) levels (f_(3/2)u_ and e_(5/2)u_) to the lowest unoccupied level (e_(5/2)g_ LUMO) of PtF_6_ (Table S3.4). The longer‐wavelength band centered at 436 nm accounts for the reddish color and the observed photo‐decomposition of PtF_6_ under the *λ*=470 nm irradiation to yield PtF_4_. Further low‐energy HOMO‐LUMO excitations give rise to additional weak absorptions down to the near‐IR (Table S3.1).[^23^, ^26^]

**Figure 5:**
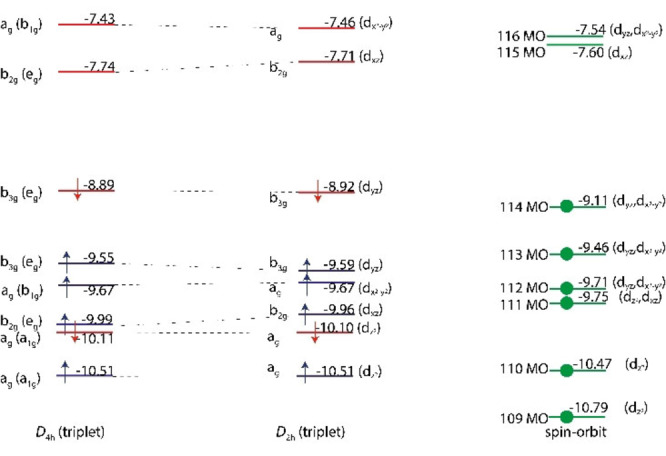
Effect of JT distortion (*D*_4h_→*D*_2h_, left) and spin–orbit coupling (right) on the orbital splitting of square planar PtF_4_. Note that α (blue) and β (red) orbitals are not energetically degenerate in the unrestricted triplet states calculation including spin polarization. The code used at the 2c level does not allow symmetry assignment of the spinors at Kramers’ unrestricted levels. The characterization of the spinors (right) has been guided by relation to the scalar‐relativistic MO assignments.

For PtF_4_ the lowest‐energy singlet and triplet configurations were evaluated at 1c‐X2C and 2c‐X2C levels. At both levels, the JT distorted open‐shell triplet configuration (^3^B_2g_) of *D*_2h_ symmetry (Figure 3) was found to be slightly lower in energy than the square‐planar closed‐shell ^1^A_1g_ state (*D*_4h_, Table S4.2). Generally, JT interactions (of electrostatic origin) and SOC (of relativistic origin) compete with each other,^[25b]^ and SOC reduces the singlet‐triplet energy gap for PtF_4_ considerably from 56 kJ mol^−1^ in the spin‐free 1c‐X2C computation to about 11 kJ mol^−1^ in the two‐component 2c‐X2C computations (TPSSh level, Table S4.2). However, in contrast to PtF_6_, in which SOC completely suppresses the expected JT distortion, the JT distortion of PtF_4_ in its lowest‐energy triplet state is only partly quenched by SOC. The effect of JT distortion and SOC on the platinum d orbital splitting of planar PtF_4_ is illustrated in Figure 5. The *D*_4h_→*D*_2h_ distortion of the triplet state increases the gap between the highest occupied and the lowest unoccupied MOs, while this gap is further increased by SOC. As expected, the experimentally observed splitting of the degenerate e_u_ stretching fundamental of PtF_4_ (*D*_4h_) into two (Pt−F) stretching modes (b_2u_+b_3u_) with similar intensities is predicted at both levels, the 1c‐ and the 2c computations. While this mode splitting is consistently>10 cm^−1^ in the scalar‐relativistic 1c‐X2C calculation using different DFT functionals, it is reduced significantly by the inclusion of SOC (Table S4.3) to values close to the experimentally observed frequencies (Table 1).

Apart from the splitting of the degenerate fundamentals, predicted IR frequencies for the lowest‐energy ^3^B_2g_ and ^1^A_1g_ states of PtF_4_ are rather similar (Table 1), reflecting the small distortion in the triplet ground state from *D*_4h_ symmetry (Figure 3). Due to their different spin multiplicity, however, the two states can be easily distinguished by their different UV/Vis spectra. For the triplet state, four low‐energy CT excitation bands are predicted at the 1c‐ZORA TDDFT level and their number increases up to eight CT excitations in the range of 250–370 nm when SOC is taken into account at the 2c‐ZORA TDDFT level (Tables S4.7 and S4.8). As expected, the UV/Vis spectrum predicted for the closed‐shell singlet PtF_4_ (^1^A_1g_, *D*_4h_) is much simpler, and only two twofold degenerate UV transitions are predicted at the 2c‐X2C TDDFT level using different functionals (Table S4.9). With excitation wavelengths of 288 and 314 nm, the former transition is predicted to be about 3.4 times stronger than the latter (TDDFT 2c‐X2C TPSSh result, Table S4.9). These computed low‐energy CT transitions of singlet PtF_4_ (^1^A_1g_, *D*_4h_) agree well with the experimentally observed transitions at *λ*_max_=272 and 325 nm (Figures 4 and S2.6). Hence, based on the UV/Vis spectrum we must consider a second closed‐shell isomer of PtF_4_ with *D*_4h_ symmetry, simultaneously formed by the photo‐destruction of PtF_6_ and co‐existing with the lower‐energy triplet isomer in the solid neon matrix.

The UV/Vis spectrum of PtF_4_ is clearly dominated by transitions of the singlet isomer. This is particularly true for the vibrationally resolved excitation at shorter wavelengths, which is predicted to be more intense. On the other side, the higher intensity of the experimental transition around 325 nm (Figures 4 and S2.6) can probably be attributed to a superposition of excitations of both isomers. The singlet isomer, on the other hand, is difficult to detect in the experimental IR spectrum. Because of similar bond lengths and vibrational frequencies of these isomers, the strong degenerate Pt−F stretching band of the minor isomer is predicted within the region of the two closely spaced stretching bands of the triplet isomer, and the much weaker deformation bands of the minor isomer (Tables 1 and S4.6) are difficult to detect experimentally anyway.

Even if the solid matrix confinement and host‐guest interactions in a Ne‐matrix site at 6 K prevent a structural rearrangement from *D*_4h_ to *D*_2h_ symmetry, it cannot be ruled out that the DFT calculations overestimate the energy difference of these spin isomers. Thus, the combination of JT distortion and SOC leads to two co‐existing configurations with different magnetic spin states (magnetic bistability) for molecular PtF_4_. The considerable SOC effects observed for the fluorides PtF_6_ and PtF_4_ are remarkable. It has also been shown that the strength of SOC in related platinum complexes depends on the nature of the ligands. Computational studies on the dianions PtX_6_^2−^ (X=F, Cl)[^18^, ^28^] and PtX_4_^2−^ (X=F, Cl, Br)^[19]^ have shown that SOC effects are most dramatic in the fluorides (X=F). This is caused by the high platinum 5d orbital dominance of the corresponding partially occupied π* MOs in the fluorides and due to the redox‐innocent character of the fluorine ligand in these complexes.

The experimental observations in the solid Ar matrix suggest a stepwise elimination of F atoms from PtF_6_ via PtF_5_ to yield PtF_4_ according to the Equations (1) and (2), where both steps can be triggered successively by selective irradiations. In contrast to this, the PtF_5_ intermediate was not detected during the photo‐decomposition of PtF_6_ in the Ne matrix. It can, however, not be ruled out that the F atom initially formed in solid neon by Pt−F bond cleavage [Eq. (1)] during irradiation abstracts a second F ligand from the PtF_5_ intermediate and reacts irreversibly to PtF_4_+F_2_ [Eq. (2)], and that a multistate process is involved.

(1) $$\mathrm{PtF}{}_{6}\overset{h\nu }{\underset{}{↔}}\mathrm{PtF}{}_{5}+F$$

(2) $$\mathrm{PtF}{}_{5}+F\overset{h\nu }{\underset{}{\to }}\mathrm{PtF}{}_{4}+F{}_{2}$$

From a purely ground‐state thermochemical perspective, process **2** is clearly feasible (Table 3), as addition of a fluorine atom to PtF_5_ to form PtF_6_ (Table 3, reaction 5) generates more free energy than needed for subsequent F_2_ elimination (Table 3, reaction 9).

**Table 3:** Reaction enthalpies (Δ*H*) and free energies (Δ*G*) (calculated for *T*=5 K and *p*=1 bar) for the F atom and F_2_ addition to PtF_*n*_ (*n*=2–6)^[a]^

Reaction	Δ*H* [kJ/mol]	Δ*G* [kJ/mol]
1. PtF+F→PtF_2_	−424.4	−424.1
2. PtF_2_+F→PtF_3_	−214.0	−213.7
3. PtF_3_+F→PtF_4_	−285.7	−285.3
4. PtF_4_+F→PtF_5_	−120.0	−119.7
5. PtF_5_ + F→PtF_6_	−181.5	−181.2
6. PtF+F_2_→PtF_3_	−490.9	−490.6
7. PtF_2_+F_2_→PtF_4_	−352.2	−351.9
8. PtF_3_+F_2_→PtF_5_	−258.3	−257.9
9. PtF_4_+F_2_→PtF_6_	−154.1	−153.7

[a] Electronic energies have been obtained at the 2c‐X2C‐B3LYP level.

### Photochemistry of PtF_4_

**Figure 6:**
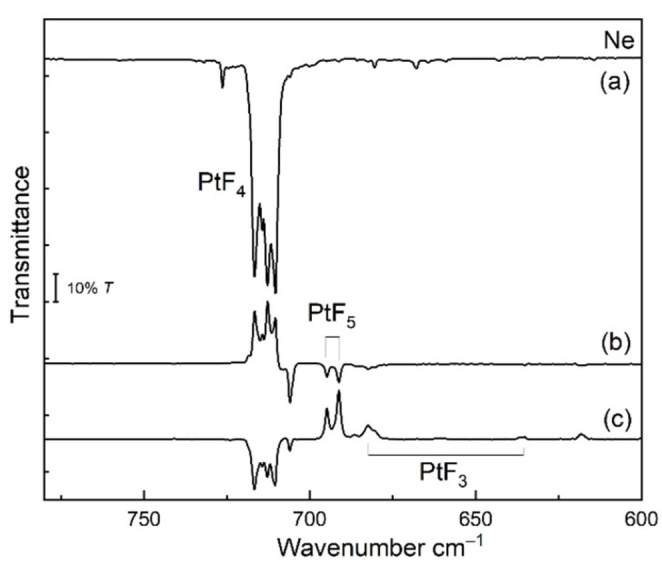
Infrared matrix‐isolation spectra obtained in solid neon at 6 K. a) IR recorded after *λ*=470 nm (blue LED) irradiation of the initial deposit of the PtF_6_ precursor seeded in excess Ne. The difference spectra (b) and (c) show spectral changes obtained after *λ*=266 nm (UV laser) irradiation for 60 min and subsequent *λ*=375 nm (UV LED) irradiation for 80 min, respectively. Downward‐pointing bands in the difference spectra are formed at the expense of upward‐pointing bands.

As the chemistry of the molecular platinum fluorides is still largely unknown, we carried out additional experiments to explore the photochemistry of PtF_4_ and the reaction of IR laser‐ablated platinum atoms with elemental fluorine gas diluted in the rare gases neon and argon. For irradiation of PtF_4_ we used a *λ*=266 nm laser to avoid a simultaneous excitation of the broad PtF_6_ CT bands. However, mainly the strong Pt−F stretching bands of PtF_6_ and PtF_5_ increased at the expense of the PtF_4_ bands under the 266 nm radiation. Analogous to the above described Ar‐matrix spectrum (Figure S2.4), for molecular PtF_5_ only the intense degenerate e‐type Pt−F stretching band at 691.2 cm^−1^ (accompanied by a weaker matrix site at 694.8 cm^−1^) could be safely assigned in this experiment (Table 2). In addition, much weaker Pt−F stretching bands appeared at 682.4, 635.3 and 617.9 cm^−1^ (Figure 6). Similar results were obtained with *λ*=254–400 nm broad‐band UV or 254±5 nm radiation (Figure S2.7). These observations suggest a photo‐dissociation of F_2_ molecules rather than a Pt−F bond cleavage of PtF_4_, followed by a stepwise fluorination of PtF_4_ to PtF_6_ (reactions 4 and 5, Table 3). These experiments support our previous assignment of the PtF_5_ stretching band and provide Pt−F band positions for the hitherto unknown molecular PtF_3_ (Tables 2 and S7.5). For PtF_3_ in its ^2^A_2_ ground state a T‐type structure with one shorter and two longer Pt−F bonds has been computed at the 2c‐X2C DFT level (Figure 3, Table S7.4). Photo‐dissociation of F_2_ is also efficient under *λ*=375±10 nm UV–LED radiation and the F‐atoms thus formed react with PtF_5_ and PtF_3_ back to PtF_4_ and traces of PtF_6_ (Figure 6c). Because of their limited mobility in a solid neon matrix, F‐atoms are very efficient fluorinating agents under matrix isolation conditions.^[29]^ The addition of a fluorine radical is strongly exothermic for the lower platinum fluorides (Table 3) and is expected to proceed without activation barrier.

**Figure 7:**
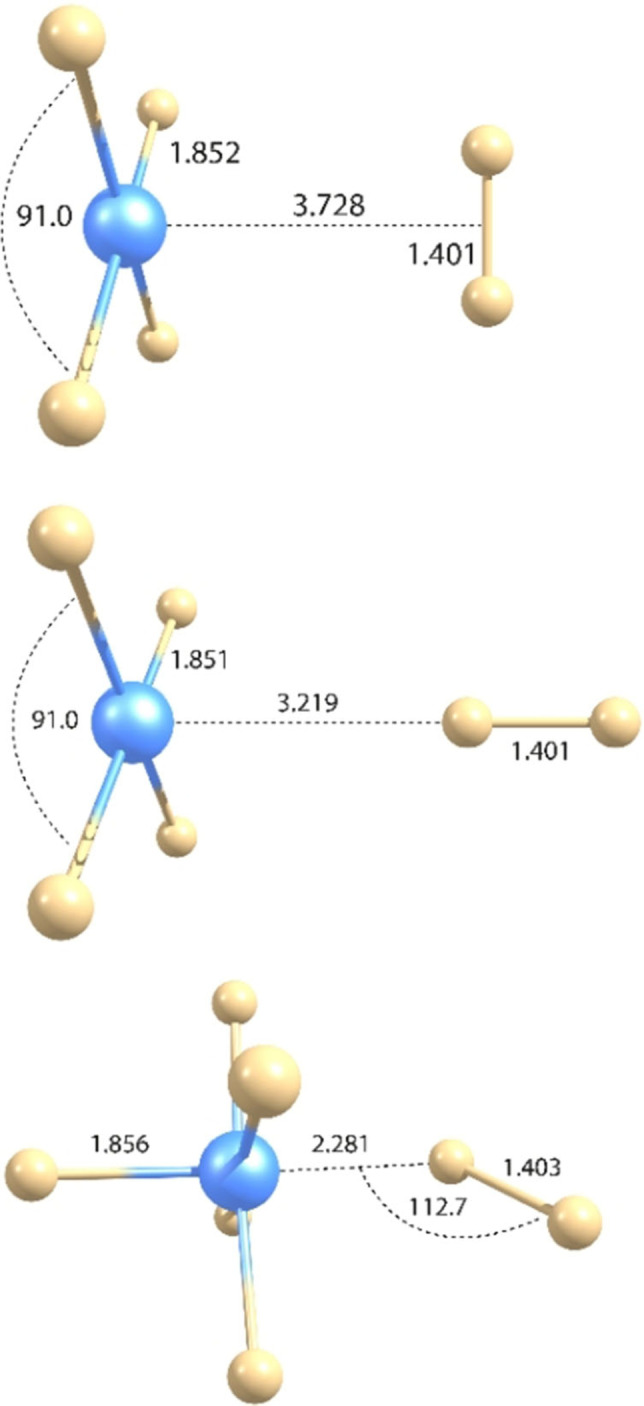
Computed structures of the difluorine complexes PtF_*n*_⋅F_2_, *n*=4, 5. For *n*=4, the 2c‐X2C TPSSh‐D3 level was used, for *n*=5, the B3LYP‐D3 level, as TPSSh‐D3 leads to dissociation of F_2_. Bond lengths are given in Å and angles in degrees.

These results suggest that the photochemistry of the system described is dominated by the photo‐dissociation of F_2_ and F‐atom reactions rather than the photo‐destruction of molecular PtF_4_. Due to the restrictions of the matrix environment, the F_2_ fragment formed by the initial photolysis of PtF_6_ remains close to the PtF_4_ molecules or could even be trapped within the same matrix cage together with PtF_4_. In this latter case a PtF_4_⋅F_2_ complex could possibly be formed that reacts under near‐UV photolysis back to PtF_6_. This led us to compute PtF_4_⋅F_2_ complexes in both side‐on and end‐on coordination to platinum (Figure 7), comparing the lowest triplet and singlet states. The interaction energies for these complexes are small. They are dominated by dispersion interactions (for details see the Supporting Information), consistent with weak van‐der‐Waals complexes, and the side‐on complex was found to be somewhat lower in energy at all levels (Table S6.1). Computed structural and vibrational data for the side‐on and end‐on PtF_4_⋅F_2_ complexes, respectively, at various 1c‐ and 2c‐levels (Tables S6.2–S6.5) show only small vibrational frequency shifts (by <3 cm^−1^ at 2c‐X2C level using the TPSSh functional and D3 dispersion corrections) compared to free PtF_4_, which corroborates the relatively weak interactions.

Results for PtF_4_⋅F_2_ and the previously detected AuF_5_⋅F_2_ (AuF_7_) complex^[13]^ prompted us also to consider complexes of PtF_5_ with F_2_. In contrast to PtF_4_, an end‐on bent *C*_s_‐symmetrical coordination with a F−Pt−F angle of around 112–115° (Figure 7, Table S6.6) has been found to be energetically favored over a *C*_2v_‐symmetrical side‐on coordination in this case (by about 37 kJ/mol at B3LYP 1c‐X2C level). Here the larger interaction is not anymore dominated by dispersion contributions. Indeed, optimization at TPSSh‐D3 level leads to dissociation of the F−F bond. While the interaction is thus more pronounced, the spectra provided no evidence for the formation of this complex.

### Reaction of Pt atoms with fluorine

**Figure 8:**
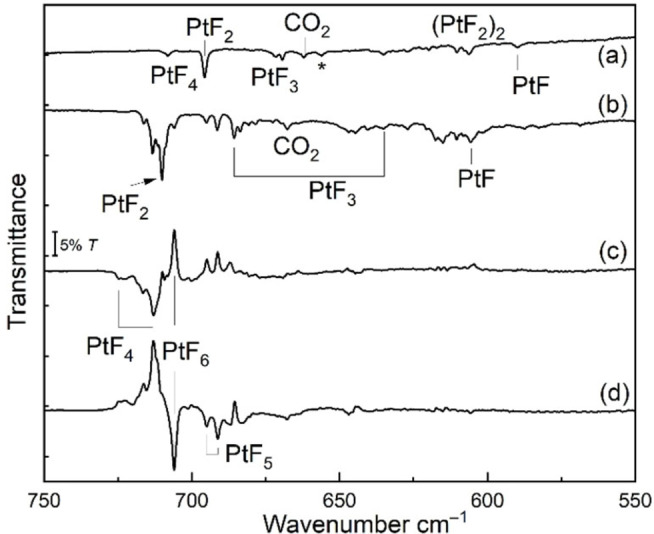
Infrared spectra obtained from the reaction products of laser‐ablated Pt atoms with F_2_ after co‐deposition for 60 min seeded a) in excess Ar at 10 K, and b) in excess Ne at 5 K. Difference spectra (see legend to Figure 6) obtained from spectra recorded before and after c) *λ*=470 nm (blue LED) irradiation for 30 min, and d) subsequent *λ*=278 nm (UV LED) irradiation for 35 min.

The initially formed product of the exothermic reaction between laser‐ablated platinum atoms and F_2_ is expected to be the linear PtF_2_.^[11]^ Due to a low concentration of F_2_ gas diluted in the corresponding noble gas (1 : 1000–1 : 200) and, owing to the short reaction time prior to solidification of the deposit on the matrix support, the formation of higher platinum fluorides is suppressed. However, fluorine atoms are also obtained from the noble gas/fluorine stream by dissociation of F_2_ within the hot plasma plume and also by the broad‐band radiation produced by the laser ablation of metals.^[30]^ These fluorine atoms allow for a successive fluorination of the initially formed Pt atoms and of PtF_2_ during condensation of the gas mixture on the cold matrix support. Although the noble gases neon and argon are considered to be inert matrix hosts, they differ in their specific host–guest interactions and often also in terms of the observed reaction products. In fact, infrared spectra of the reaction products of laser‐ablated Pt atoms with F_2_ in neon and argon matrices (Figure 8) appear rather different at first glance, and the Ar‐spectrum of the Pt−F stretching region is much simpler. This is partly because of stronger argon–guest interactions, which give rise to significant neon‐to‐argon matrix‐shifts, particularly for the lower platinum fluorides PtF (Δ*ν*=−15.6 cm^−1^) and PtF_2_ (Δ*ν*=−14.5 cm^−1^) compared to a much smaller shift of the strong PtF_6_ stretching band of <4 cm^−1^ (Table 2). Substantial neon‐to‐argon matrix shifts have also been observed for AuF (Δ*ν*=+18.7 cm^−1^, ArAuF in solid Ne compared to NeAuF) and AuF_2_ (Δ*ν*=−24.7 cm^−1^ for pure Ar and Ne matrices, respectively).^[31]^

In the Ne spectrum (Figure 8b–d), the intense IR bands of the species with linear Pt−F bonds (PtF_2_, PtF_4_, and PtF_6_) are grouped together in the spectral region between 705–717 cm^−1^ (Figure 8b–d). However, their assignment is made possible by their different photo‐behaviors. Interestingly, the higher platinum fluorides PtF_5_ and PtF_6_ were not observed in laser ablation experiments using argon as a host gas (Figure 8a). This can probably be explained by the slower condensation rate of neon (25 K f.p.) on a 6 K surface compared to that for argon (84 K f. p.),^[32]^ leaving more time for the successive fluorination of the initially formed PtF_2_ during condensation of neon gas onto a solid matrix. Fluorination by photo‐mobilized F atoms is also more efficient in solid neon than in solid argon. A list of all observed Pt−F stretching frequencies for the observed PtF_*n*_ (*n*=1–6) species is given in Table 2, where they are compared to computed frequencies at the one and two‐component X2C‐TPSSh level, from which the influence of SOC effects on these frequencies can be evaluated. We note that the computed Pt−F bond lengths of PtF_2_ (1.837 Å), PtF_4_ (^3^B_2g_: 1.852 Å) and PtF_6_ (1.861 Å, Figure 3) increase slightly within this series. This trend correlates well with computed frequencies for the antisymmetric stretching modes of their linear F−Pt−F units (PtF_2_: 710 cm^−1^; PtF_4_ (^3^B_2g_) averaged value: 705 cm^−1^; PtF_6_: 698 cm^−1^, 2c‐X2C‐TPSSh results, Table 2), and there is an excellent agreement between these computed and the experimentally observed frequencies in the solid Ne matrix: PtF_2_: 710 cm^−1^, PtF_4_ (^3^B_2g_): 715 cm^−1^ (average value), and PtF_6_: 706 cm^−1^ (Table 2), considering that the largest matrix‐shift compared to the gas‐phase value is expected for the low‐valent PtF_2_ species.

Computed natural (NPA) atomic charges and Pt‐orbital populations (Table S7.6) indicate decreasing charge on the fluorine atoms within the series PtF_2_ (−0.525), PtF_4_ (^3^B_2g_, −0.456) and, PtF_6_ (−0.384, 2c‐X2C‐B3LYP), and they follow the same trend on average for PtF_3_ and PtF_5_. As previously discussed for other examples,^[33]^ the computed atomic charges of platinum show the expected correlation with its formal oxidation numbers (Figure S2.9). While the computed natural electron population of the 5d orbitals of PtF_2_ (8.03, Table S7.6) corresponds well to a formal d^8^ configuration of a Pt^II^ compound, that of PtF_4_ (5d population: 7.57) and PtF_6_ (7.24) differ significantly from a formal d^6^ (Pt(IV)) and d^4^ (Pt(VI)) configuration. In addition, these calculations also reveal significant 6 s‐occupations, particularly for PtF_2_ (0.88, Table S7.6), thus indicating 5d, 6s‐hybridization. Thus, the fully ionic approximation appears to be a worse bonding model for these binary platinum fluorides, for which the degree of covalency increases significantly along the series.

## Conclusion

We have presented a systematic spectroscopic study of the series of molecular platinum fluorides PtF_*n*_ (*n*=1–6) and, for the first time, vibrational frequencies of the molecules PtF_3_, PtF_4_ and PtF_5_. These species were prepared by a photo‐initiated defluorination of PtF_6_ and the reaction of fluorine atoms or F_2_ molecules with laser‐ablated platinum atoms, respectively, and were isolated under cryogenic conditions in rare‐gas matrices. The platinum fluorides produced in the laser‐ablation experiments depend on the noble‐gas host. However, it was found that the formation of PtF_4_ by blue‐light (*λ*=470 nm) irradiation of PtF_6_ is almost quantitative in both solid neon and argon. PtF_5_ and PtF_3_ were formed simultaneously by subsequent UV irradiation of PtF_4_. The assignment of their vibrational spectra is supported by one‐ and two‐component quasirelativistic DFT computations, which account for scalar relativistic (SR) and SOC effects. Computations at the lowest‐energy triplet and singlet surfaces of PtF_4_ show competing JT and SOC effects, which result in a magnetic bistability with the co‐existence of ^3^B_2g_ and ^1^A_1g_ electronic states having *D*_2h_ and *D*_4h_ symmetry, respectively. The presence of both of these states in the solid neon matrices has been verified spectroscopically: five fundamental and two combination bands of the lowest‐energy triplet state of PtF_4_ were assigned in its IR spectrum, whereas the UV/Vis spectrum is dominated by the CT bands of singlet PtF_4_. Although further calculations predict structures and IR spectra of PtF_4_ and PtF_5_ complexes with F_2_ at 1c‐ and 2c‐DFT levels using different functionals, such difluorine complexes could not be assigned in the experimental spectra.

## Conflict of interest

The authors declare no conflict of interest.

## Supporting information

As a service to our authors and readers, this journal provides supporting information supplied by the authors. Such materials are peer reviewed and may be re‐organized for online delivery, but are not copy‐edited or typeset. Technical support issues arising from supporting information (other than missing files) should be addressed to the authors.

Supporting Information
