# Supplementary material for: Matrix Isolation Spectroscopic and Relativistic Quantum Chemical Study of Molecular Platinum Fluorides PtF n (n=1–6) Reveals Magnetic Bistability of PtF4
Source: Chemistry. 2021 Aug 19;27(54):13642–50. doi: 10.1002/chem.202102055 (PMC8518493; doi:10.1002/chem.202102055)
Supplement: Supplementary file 1 — Supporting Information [file CHEM-27-13642-s001.pdf]

Chemistry–A European Journal

Supporting Information

Matrix Isolation Spectroscopic and Relativistic Quantum Chemical Study of Molecular Platinum Fluorides PtF_n ($n = 1–6$) Reveals Magnetic Bistability of PtF_4

Gene Senges[+], Lin Li[+], Artur Wodyński[+], Helmut Beckers, Robert Müller, Martin Kaupp,* and Sebastian Riedel*

Contents

Part 1. Experimental and Computational Details

1.1. Experimental details 2

1.2. Computational details 2

1.3. References to Part 1 3

Part 2. Supporting Figures

Figures S2.1–S2.9 5–9

Part 3. Structure, vibrational frequencies and electronic transitions of PtF_6

Tables S3.1–S3.4 10–14

Part 4: Structures, vibrational frequencies and electronic transitions of PtF_4

Tables S4.1–S4.9 15–23

Part 5: Computed structures and vibrational spectra of PtF_5 ($^2B_2$, C_{4v})

Tables S5.1–S5.2 24–26

Part 6: Computed structures and vibrational frequencies of difluorine complexes $PtF_n{\cdot}F_2$, n = 4, 5

Tables S6.1–S6.8 27–37

Part 7: Computed structures and vibrational spectra of PtF_n, n = 1–3

Tables S7.1–S7.6 38–41

Part 8 Additional scalar-relativistic DFT calculations on PtF_n (n = 1–6) and PtF_4 complexes

Table S8.1 42–43

Part 9 Scalar-relativistic CCSD(T) calculations on PtF_n (n = 1–5)

Table S9.1 44–45

Part 1. Experimental and Computational Details

1.1 Experimental details

Platinum hexafluoride was prepared according to the original protocol of Weinstock *et al*.[1] A platinum wire (0.5 mg) was burning in cold F_2 atmosphere (3 bar) in one passivated stainless reactor (volume = 54.5 ml) by electric ignition. The reactor was in a –196 °C liquid nitrogen bath. The product PtF_6 was trapped by liquid nitrogen and stored in fluoroplastic (PFA) tube equipped with a stainless-steel valve. To avoid the contamination of lower platinum fluorides, a high excess ratio of fluorine (Pt: F_2 = 1:10) was used. The PtF_6 sample was purified by long-time pumping and its initial purity was monitored by IR spectroscopy. After the purification, PtF_6 sample was maintain in a cooling bath at temperatures around –90 °C during deposition. The evaporated products of PtF_6 sample were subsequently co-condensed with an excess of pure noble matrix gas (Ne or Ar), which were frozen on the matrix support for further measuring (see below).

The technique of matrix-isolation infrared (IR) spectroscopy and laser-ablation apparatus have been described in previous works.[2] Excited Pt/Pd atoms, formed by laser-ablation, reacted with F_2 and co-deposited in different noble gases matrix. The Nd:YAG laser (1064 nm, 10 Hz) apparatus with a pulse energy of up to 50 mJ/cm^2 was focused onto the rotating metal target. Diluted F_2 (0.05–1%) reacted with hot excited metal atoms formed by the laser, which mixed with Ne or Ar matrix gases and all co-deposited on a cold gold-plated mirror (6–12 K). During their deposition, a closed-cycle liquid helium refrigerator (Sumitomo Heavy Industries, RDK-205D) was used to cool down the self-made vacuum chamber. IR and FIR spectra were recorded at a resolution of 0.5 cm^{-1} on a Bruker Vertex 80v spectrometer, by using MCT detector (4000–450 cm^{-1}) and liquid helium cooled bolometer (680–180 cm^{-1}), respectively. UV/Vis (resolution = 0.1 nm) spectra of the platinum fluorides were recorded on a Perkin Elmer Lambda 850+ UV/Vis Spectrophotometer. Matrix samples were annealed to different temperatures and irradiated by using various LED sources (OSLON 80 4+ PowerStar Circular 4 LED Arrays: λ = 528±30 nm (True Green), λ = 470±20 nm (Blue), λ = 455±20 nm (Deep Blue), λ = 375±10 nm (Qioptiq ML3 UV LED), and λ = 278nm (100mW, AMPYR LED33UV278-6060-100), as well as a pulsed λ = 266 nm Q-switched solid-state laser (CryLas 6FQSS266-Q2-OEM, 266/532 nm, 0.8 µJ @10kHz).

1.2 Computational details

One and two-component X2C[3] DFT computations[4] have been performed using Turbomole 7.5.0[5] with x2c-TZVPall-2c[6] basis sets and integration grid m5. Two-electron spin-orbit terms were included using the scaled-nuclear-spin-orbit (SNSO)[7] approach in its original parameterization by Böttcher.[8] B3LYP,[9] PBE0[10] and TPSSh[11] hybrid functionals have been compared. SCF convergence was obtained up to 10^{-8} a.u.. Weight derivatives of grid points have been included during structure optimization and vibrational frequency calculations. The 1c-X2C calculations exploited point-group symmetries D_{2h} (PtF_2, PtF_4), C_{2v} (PtF, PtF_3, PtF_5, $PtF_4{\cdot}F_2$) and C_s ($PtF_5{\cdot}F_2$). No point-group symmetry has been used in the 2c calculations (time reversal symmetry was used for PtF_6, the 2c analogue of the restricted Kohn-Sham approach). Structure optimizations were converged to 10^{-4} in the gradient norm. Numerical second derivatives of analytical gradients were used to calculate vibrational frequencies, with a differentiation increment of 0.02 Bohr. Dispersion corrections were included using Grimme's DFT-D3[12] scheme (in one case DFT-D4[13] has been used for comparison) also with Becke-Johnson (BJ) damping[14] and with the VV10[15] van-der-Waals functional for the ωB97M-V[16] functional at 1c level. Computations of UV/Vis spectra at 2c-X2C level were only possible for PtF_6, as the 2c-TDDFT[17] module in Turbomole is currently restricted to closed-shell (Kramers restricted) ground states.

Additionally, one- and two-component DFT computations of selected systems (PtF_4 and PtF_6) have also been carried out with the quasirelativistic zero-order regular approximation (ZORA)[18] using the ADF2017[19] package. These calculations used TZ2P[20] Slater-type-orbital (STO) basis sets and full optimization of the core shells. B3LYP, PBE0 and TPSSh functionals have been compared. The adiabatic kernel in the TDDFT[21] calculations has been approximated by an ALDA (adiabatic local density approximation) kernel for all examined functionals and additionally with a full adiabatic GGA kernel for B3LYP and PBE0. A Hartree-Fock kernel has been included for hybrid functionals. For PtF_6 both 1c- and 2c-calculations exploited O_h symmetry for optimization, frequency calculations as well as for TDDFT calculations of excitation spectra when using the ALDA kernel. The more sophisticated use of the GGA adiabatic kernel for a given functional (*fullkernel* option involving the LibXC[22] library) did not allow the use of symmetry. The 2c triplet calculations on the PtF_4 molecule also employed C_1 symmetry and used a non-collinear potential. An approximate unrestricted TDDFT approach within the Tamm-Dancoff approximation (TDA) was used to compute the UV/Vis spectrum of PtF_4. Additional 1c-TDA and full 1c-TDDFT calculations were done for comparison. As a meta-GGA kernel is so far not available, TPSSh TDDFT results are reported only for an ALDA kernel.

As reference data for the DFT computations, additional structure optimizations and numerical harmonic vibrational frequency calculations for the first five members of the PtF_n series (n = 1–5) have also been performed at the 1c coupled cluster CCSD(T) level, using augmented correlation consistent triple-ζ basis sets for fluorine (aug-cc-pVTZ)[23] and the corresponding aug-cc-pVTZ-PP[24] basis with small-core relativistic pseudopotential for platinum.[23] All coupled cluster calculations have been done with the Molpro19 program.[25]

1.3 References to Part 1

[1] B. Weinstock, H. H. Claassen, J. G. Malm, *J. Am. Chem. Soc.* **1957**, *79*, 5832.

[2] a) L. Li, H. Beckers, T. Stüker, T. Lindič, T. Schlöder, D. Andrae, S. Riedel, *Inorg. Chem. Front.* **2021**, *8*, 1215–1228; b) L. Li, A. K. Sakr, T. Schlöder, S. Klein, H. Beckers, M.-P. Kitsaras, H. V. Snelling, N. A. Young, D. Andrae, S. Riedel, *Angew. Chem. Int. Ed.* **2020**, 6391–6394;

[3] a) M. K. Armbruster, F. Weigend, C. van Wüllen, W. Klopper, *Phys. Chem. Chem. Phys.* **2008**, *10*, 1748; b) A. Baldes, F. Weigend, *Mol. Phys.* **2013**, *111*, 2617–2624;

[4] D. Peng, N. Middendorf, F. Weigend, M. Reiher, *J. Phys. Chem.* **2013**, *138*, 184105.

[5] S. G. Balasubramani, G. P. Chen, S. Coriani, M. Diedenhofen, M. S. Frank, Y. J. Franzke, F. Furche, R. Grotjahn, M. E. Harding, C. Hättig, A. Hellweg, B. Helmich-Paris, C. Holzer, U. Huniar, M. Kaupp, A. Marefat Khah, S. Karbalaei Khani, T. Müller, F. Mack, B. D. Nguyen, S. M. Parker, E. Perlt, D. Rappoport, K. Reiter, S. Roy, M. Rückert, G. Schmitz, M. Sierka, E. Tapavicza, D. P. Tew, C. van Wüllen, V. K. Voora, F. Weigend, A. Wodyński, J. M. Yu, *J. Phys. Chem.* **2020**, *152*, 184107.

[6] P. Pollak, F. Weigend, *J. Chem. Theory Comput.* **2017**, *13*, 3696–3705.

[7] Y. J. Franzke, N. Middendorf, F. Weigend, *J. Chem. Phys.* **2018**, *148*, 104110.

[8] J. C. Boettger, *Phy. Rev. B* **2000**, *62*, 7809–7815.

[9] C. Lee, W. Yang, R. G. Parr, *Phys. Rev. B* **1988**, *37*, 785–789.

[10] a) J. P. Perdew, M. Ernzerhof, K. Burke, *J. Chem. Phys.* **1996**, *105*, 9982–9985; b) C. Adamo, V. Barone, *J. Chem. Phys.* **1999**, *110*, 6158–6170;

[11] a) J. P. Perdew, S. Kurth, A. Zupan, P. Blaha, *Phys. Rev. Lett.* **1999**, *82*, 2544–2547; b) J. P. Perdew, J. Tao, V. N. Staroverov, G. E. Scuseria, *J. Chem. Phys.* **2004**, *120*, 6898–6911;

[12] S. Grimme, J. Antony, S. Ehrlich, H. Krieg, *J. Chem. Phys.* **2010**, *132*, 154104.

[13] E. Caldeweyher, C. Bannwarth, S. Grimme, *J. Chem. Phys.* **2017**, *147*, 34112.

[14] S. Grimme, S. Ehrlich, L. Goerigk, *J. Comput. Chem.* **2011**, *32*, 1456–1465.

[15] O. A. Vydrov, T. van Voorhis, *J. Chem. Phys.* **2010**, *133*, 244103.

[16] N. Mardirossian, M. Head-Gordon, *J. Chem. Phys.* **2016**, *144*, 214110.

[17] a) M. Kühn, F. Weigend, *J. Chem. Theory Comput.* **2013**, *9*, 5341–5348; b) M. Kühn, F. Weigend, *J. Phys. Chem.* **2015**, *142*, 34116; c) C. Holzer, W. Klopper, *J. Phys. Chem.* **2019**, *150*, 204116;

[18] a) Ch. Chang, M. Pelissier and Ph. Durand, *Phys. Scr.* **1986**, *34*, 394–404; b) E. van Lenthe, E. J. Baerends, J. G. Snijders, *J. Chem. Phys.* **1993**, *99*, 4597–4610; c) E. van Lenthe, E. J. Baerends, J. G. Snijders, *J. Chem. Phys.* **1994**, *101*, 9783–9792;

[19] a) G. te Velde, F. M. Bickelhaupt, E. J. Baerends, C. Fonseca Guerra, Van Gisbergen, S. J. A, J. G. Snijders, T. Ziegler, *J. Comput. Chem.* **2001**, *22*, 931–967; b) C. F. Guerra, J. G. Snijders, G. te Velde, E. J. Baerends, *Theor Chem Acc* **1998**, *99*, 391–403;

[20] E. van Lenthe, E. J. Baerends, *J. Comput. Chem.* **2003**, *24*, 1142–1156.

[21] a) S. J. A. van Gisbergen, J. G. Snijders, E. J. Baerends, *Compt. Phys. Comm.* **1999**, *118*, 119–138; b) F. Wang, T. Ziegler, E. van Lenthe, S. van Gisbergen, E. J. Baerends, *J. Chem. Phys.* **2005**, *122*, 204103;

[22] M. A.L. Marques, M. J.T. Oliveira, T. Burnus, *Comput. Phys. Commun.* **2012**, *183*, 2272–2281.

[23] R. A. Kendall, T. H. Dunning, R. J. Harrison, *J. Chem. Phys.* **1992**, *96*, 6796–6806.

[24] D. Figgen, K. A. Peterson, M. Dolg, H. Stoll, *J. Chem. Phys.* **2009**, *130*, 164108.

[25] a) H.-J. Werner, P. J. Knowles, G. Knizia, F. R. Manby, M. Schütz, P. Celani, W. Györffy, D. Kats, T. Korona, R. Lindh, A. Mitrushenkov, G. Rauhut, K. R. Shamasundar, T. B. Adler, R. D. Amos, S. J. Bennie, A. Bernhardsson, A. Berning, D. L. Cooper, M. J. O. Deegan, A. J. Dobbyn, F. Eckert, E. Goll, C. Hampel, A. Hesselmann, G. Hetzer, T. Hrenar, G. Jansen, C. Köppl, S. J. R. Lee, Y. Liu, A. W. Lloyd, Q. Ma, R. A. Mata, A. J. May, S. J. McNicholas, W. Meyer, T. F. Miller III, M. E. Mura, A. Nicklass, D. P. O'Neill, P. Palmieri, D. Peng, K. Pflüger, R. Pitzer, M. Reiher, T. Shiozaki, H. Stoll, A. J. Stone, R. Tarroni, T. Thorsteinsson, M. Wang, M. Welborn, *MOLPRO, version 2019.2, a package of ab initio programs*; b) H.-J. Werner, P. J. Knowles, G. Knizia, F. R. Manby, M. Schütz, *WIREs Comput Mol Sci* **2012**, *2*, 242–253; c) F. Eckert, P. Pulay, H.-J. Werner, *J. Comput. Chem.* **1997**, *18*, 1473–1483; d) P. J. Knowles, C. Hampel, H.-J. Werner, *J. Chem. Phys.* **1993**, *99*, 5219–5227; e) P. J. Knowles, C. Hampel, H.-J. Werner, *J. Chem. Phys.* **2000**, *112*, 3106–3107;

Part 2 Supporting Figures

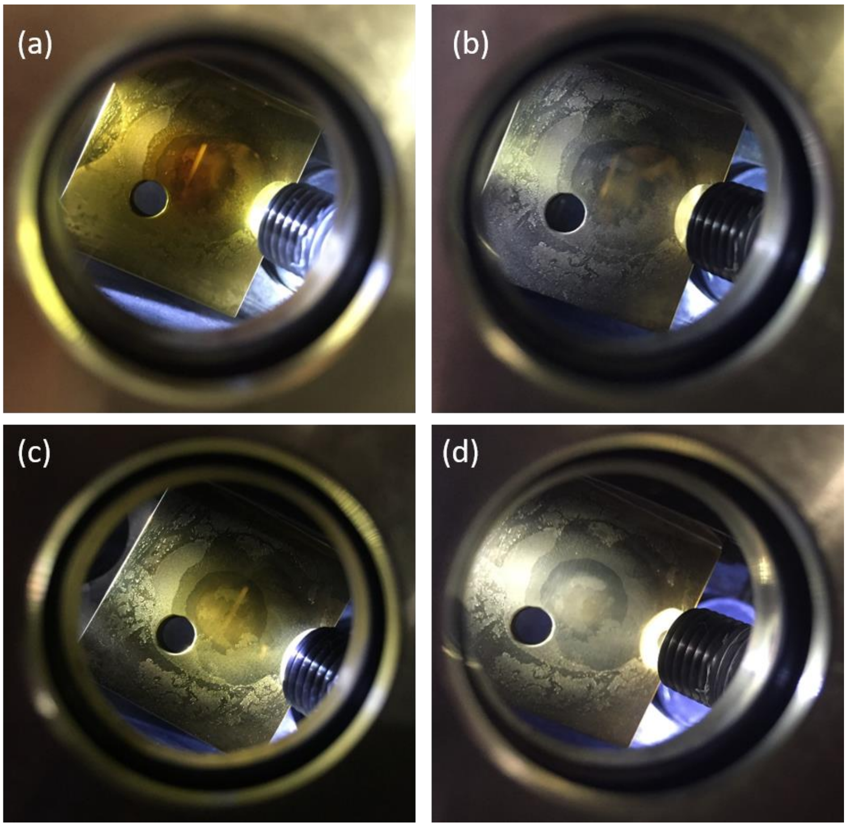

Figure S2.1. Photos of the deposited PtF_6 precursor in an excess of neon on a gold-plated matrix support taken before (**a**, **c**) and after (**b**, **d**) λ = 470 nm (blue LED) irradiation.

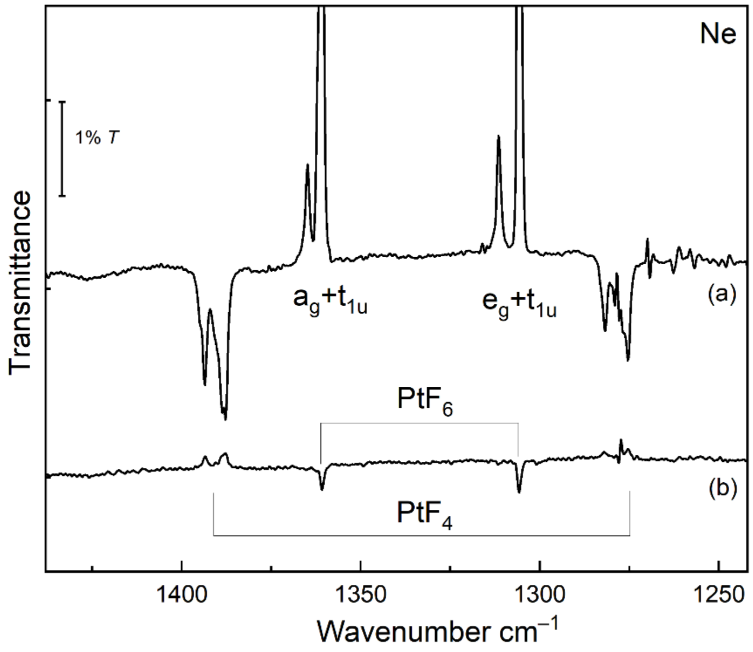

Figure S2.2. Combination bands of PtF_6 and PtF_4 recorded in a solid neon matrix. Difference spectra obtained from spectra recorded before and after **(a)** λ = 470 nm (blue LED) irradiation for 70 min, and **(b)** subsequent λ = 266 nm (Laser) irradiation for 60 min. Downward pointing bands in the difference spectra are formed at the expense of upward pointing bands.

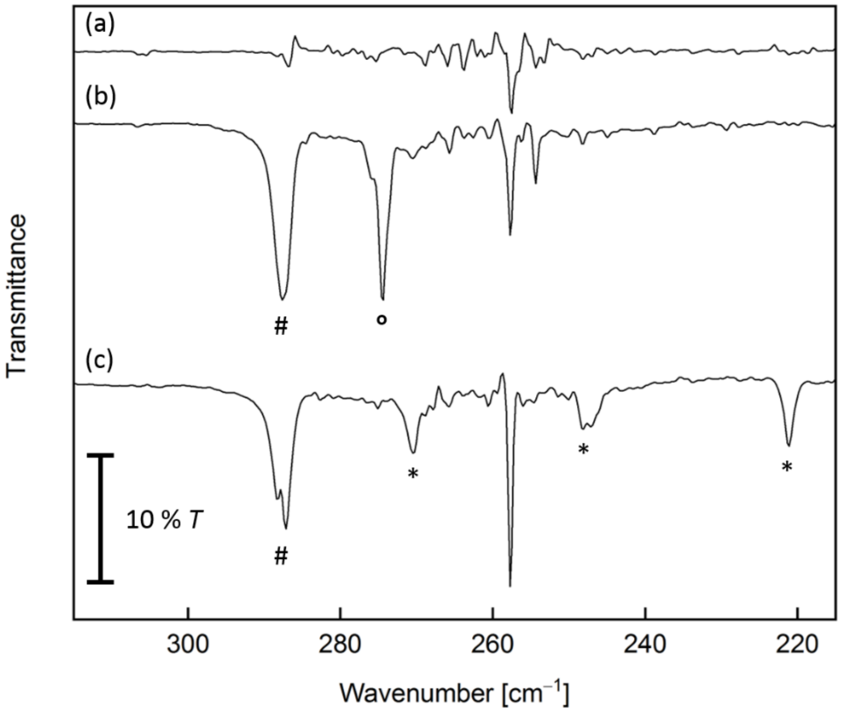

Figure S2.3 Far-IR spectra (resolution = 0.5 cm^{-1}) of PtF_6 embedded in solid neon. **(a)** Pure neon deposit (13 min deposition time). **(b)** After co-deposition of PtF_6 and neon for 30 min: the ν_4 deformation of PtF_6 at 274.4 cm^{-1} is marked by a circle and an impurity band by an octothorpe sign (#). **(c)** After subsequent irradiation with blue LED light (λ = 470 nm) for 75 min new bands appeared at the expense of the PtF_6 deformation at 270.5, 248.1 (247.1 site), and 221.1 cm^{-1}, which are marked by an asterisk and assigned to PtF_4.

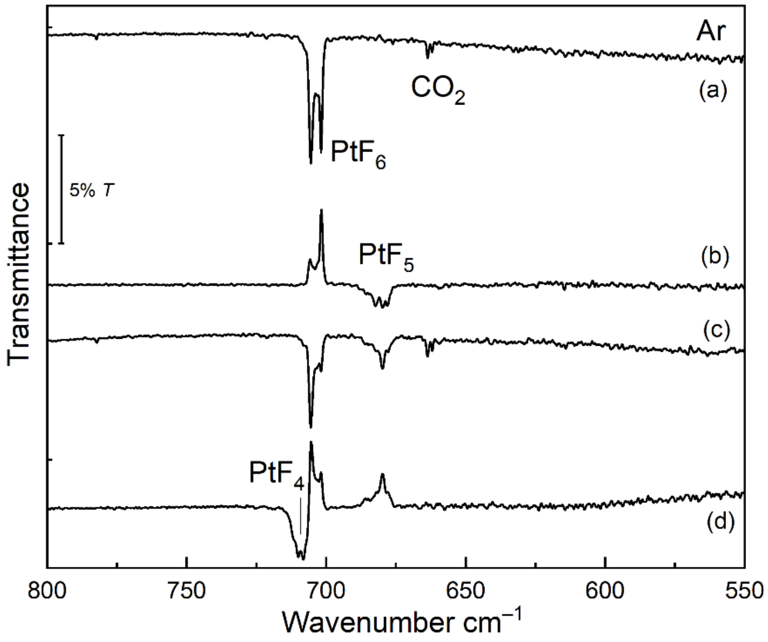

Figure S2.4. (a) Infrared Matrix-Isolation Spectra of the PtF_6 precursor seeded in excess Ar and co-deposited for 8 min at 6 K. **(b)** Difference spectrum obtained from spectra recorded before and after λ = 528 nm (green LED) irradiation for 10 min, and **(c)** transmission spectrum recorded after subsequent annealing to 15 K. **(d)** Difference spectrum obtained from spectra recorded before after λ = 455 nm (blue LED) irradiation for 10 min. Downward pointing bands in the difference spectra are formed at the expense of upward pointing bands.

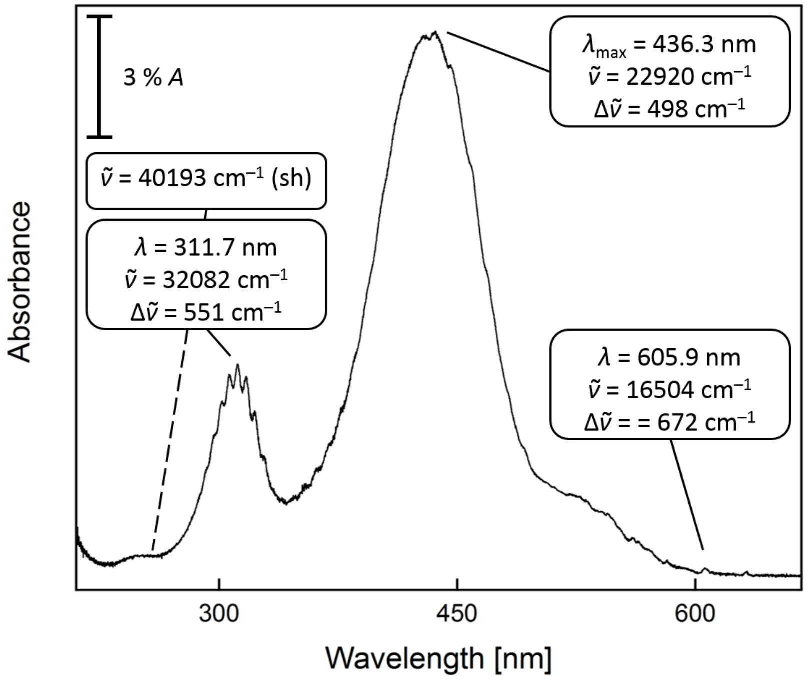

Figure S2.5 UV/Vis spectrum (resolution = 0.1 nm) recorded from a deposit (6 K) obtained from PtF_6 vapor (sample temperature between –83.1 °C to –82.4 °C) mixed with excess of neon gas. For the Mid-IR spectrum recorded from this deposit see Figure S2.8a.

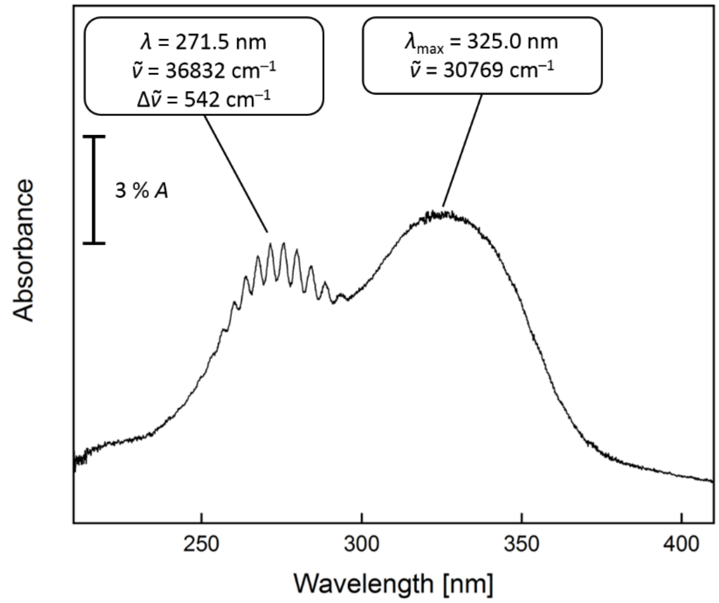

Figure S2.6 UV/Vis spectrum (resolution = 0.1 nm) of PtF_4 in solid neon at 6 K obtained after irradiation of a PtF_6/neon deposit with a blue LED (λ = 470 nm) for 75 min. For the Mid-IR spectrum recorded from this deposit see Figure S2.8b.

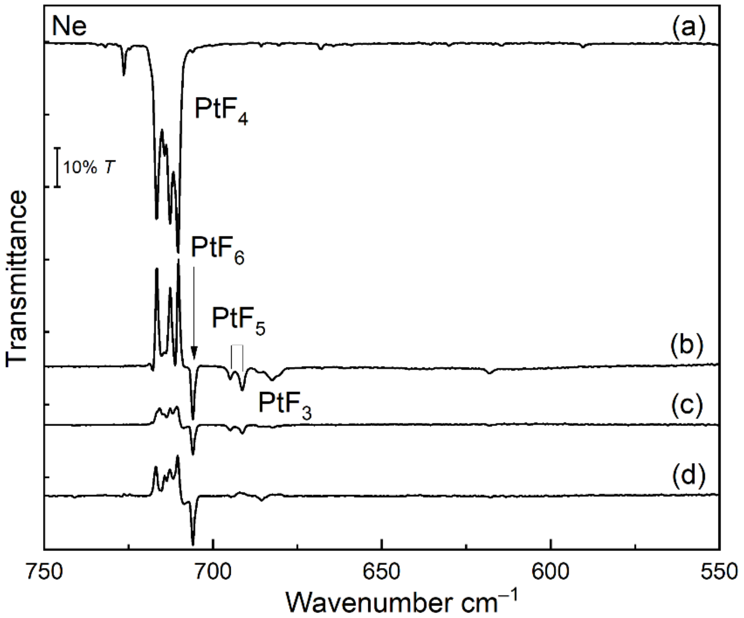

Figure S2.7. Infrared Matrix-Isolation Spectra of PtF_4 in solid neon recorded after λ = 470 nm (blue LED) photolysis of PtF_6 seeded in excess Ne **(a)**. Difference spectra obtained from spectra recorded before and after λ = 254–400 nm (Schott UV broadband filter type UG11) **(b)**, subsequent λ = 254±5 nm (bandpass filter) (**c**), and irradiation for 20 min using unfiltered medium pressure mercury arc light for 20 min **(d)**.

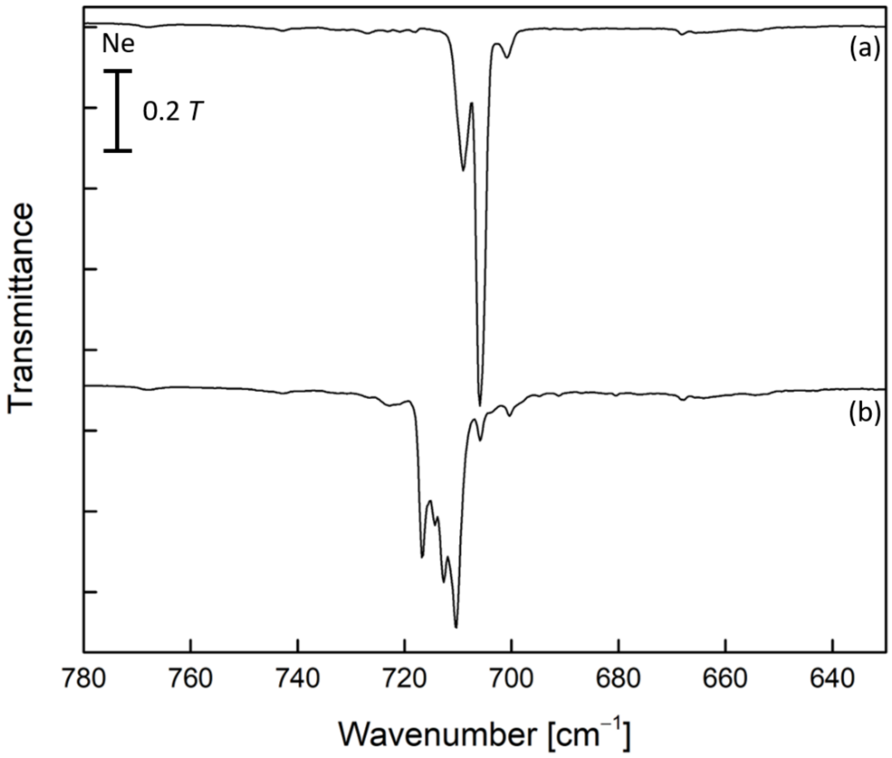

Figure S2.8. Mid-IR spectra (resolution = 0.5 cm^{-1}) of PtF_6 embedded in solid neon. **(a)** After co-deposition of PtF_6 and neon for 36 min; **(b)** after irradiation with blue LED light (λ = 470 nm) for 75 min.

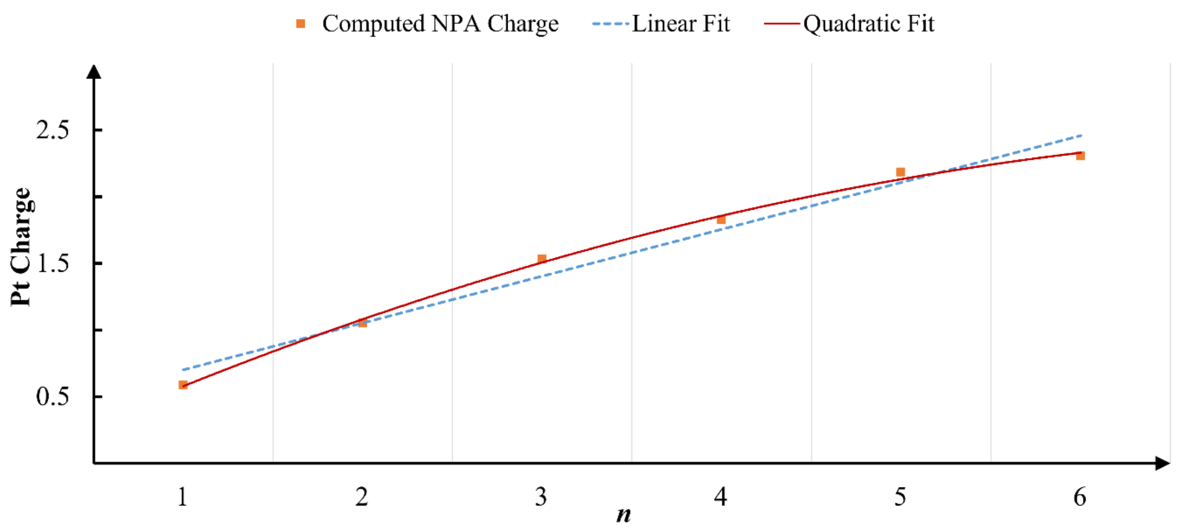

Figure S2.9. Correlation of B3LYP 2c-X2C computed NPA charges of the platinum 5d orbitals for the series of molecular binary platinum fluorides PtF_n (n = 1–6, see Table S7.6) with the platinum oxidation number n. Linear fit: broken blue line (Q(Pt) = 0.3514n + 0.3496, R^2 = 0.9715), quadratic polynomial fit: solid red line (Q(Pt) = 0.616n – 0.038n^2, R^2 = 0.9974).

Part 3. Structure, vibrational frequencies and electronic transitions of PtF_6.

Table S3.1. Electronic transitions (λ in nm) and their vibrational spacing ($\Delta\tilde{\nu}$ in cm^{-1}) obtained for PtF_6 (O_h) embedded in solid neon and N_2 matrices. Absorbance maxima are indicated by bold values.

**Ne matrix, λ [nm]** [a)]	$\Delta\tilde{\nu}$ **[cm^{-1}]** [a)]	$\Delta\tilde{\nu}_{av}$ **[cm^{-1}]** [a)]	**N_2 matrix [cm^{-1}]** [b)]
248.8 sh (40192.9 cm^{-1})		Not resolved	
286.7			
291.7	597.9		
296.5	555.0		
301.8	592.3		
306.3	486.8		
**311.7 (32082.1 cm^{-1})**	565.6	551 cm^{-1}	**32895** ($\Delta\tilde{\nu}_{av}$: 560 cm^{-1})
317.1	546.3		
322.5	528.0		
328.2	538.6		
346.8 sh			
354.3 sh	610.4		
361.3 sh	546.9		
369.0 sh	577.5		
**376.7 sh (26546.3 cm^{-1})**	554.0	581 cm^{-1}	26500 sh
385.3 sh	592.5		
394.5 sh	605.3		
403.1			
412.1	541.7		
420.0	456.6		
429.2	510.3		
**436.3 (22920.0 cm^{-1})**	379.2	498 cm^{-1}	**23500** ($\Delta\tilde{\nu}_{av}$: 555 cm^{-1})
445.8	488.4		
458.2	607.1		

468.5	479.8		
482.2	606.4		
492.0	413.1		
(506.4)	578.0		
(520.7)	542.3		
(532.3)	418.5		
(544.3)	414.2		
540.7			
560.2	643.7		
582.2	674.6		
**605.9 (16504.4 cm^{-1})**	671.8	672 cm^{-1}	16000
632.6	696.6		
2557.7 (3909.8 cm^{-1})		MIR	
3007.9 (3324.6 cm^{-1})	585.2	MIR	3300

[a]: This work, $\Delta\tilde{\nu}_{av}$: mean vibrational spacing. [b]: Ref. [26]

Computational results on PtF_6

The ground state of PtF_6 in O_h symmetry is computed to be diamagnetic at 2c level, as spin-orbit coupling splits the levels arising from the t_{2g} MOs into a fourfold degenerate $f_{(3/2)g}$ spin-orbit level below a twofold degenerate $e_{(5/2)g}$ level, and the lower of these levels is occupied by four electrons (see Figure 1, main text). Indeed, the lowest-energy excitation is between these two levels and thus reflects the SO splitting in the ground state, which is responsible for the observed diamagnetism, for the absence of a Jahn-Teller distortion in an octahedral d^4 complex, for absorptions down to the near-IR, and for the unusual NMR shifts of PtF_6[27]. The present two-component results confirm clearly that the electronic structure of PtF_6 is dominated by SO coupling and thus cannot be described properly at scalar relativistic levels,[28] which would suggest a triplet ground state. The computed IR-active Pt–F stretching frequency is only a few wavenumbers below experiment at B3LYP and TPSSh levels, while PBE0 overestimates it by a somewhat larger amount (Tables S3.2, S3.3).

In spite of the lowest-lying excitation involving the spin-orbit splitting of the t_{2g} level (see above), two important triply degenerate (T_{1u}) excitations with F-to-Pt charge-transfer character account for the observed low-energy bands in the UV/Vis spectrum (Table S3.4). They involve $f_{(3/2)u}$ and $e_{(5/2)u}$ occupied and $e_{(5/2)g}$ (LUMO) virtual orbitals (on the basis of 2c-ZORA ALDA kernel calculations). Mulliken analysis at 2c-X2C level shows that both bands come from F(p) (with small amount of Pt(p)) to LUMO Pt(5d) excitations. The longer-wavelength excitation at 427 nm accounts for the reddish color of PtF_6.

Table S3.2. Computed Pt–F bond lengths and asymmetric stretching fundamental frequencies of ground-state PtF_6 (O_h symmetry) obtained at different two-component DFT levels in comparison with experimental values.

Functional	Hamiltonian	d(Pt–F) in Å	ν_1 in cm^{-1}
B3LYP	2c-X2C	1.862	697
PBE0	2c-X2C	1.843	724
TPSSh	2c-X2C	1.861	698
B3LYP	2c-ZORA	1.862	698
PBE0	2c-ZORA	1.849	717
TPSSh	2c-ZORA	1.861	702
	Exp.	1.851, 1.850 [a)]	706[b)]

a) Ref. [29]
b) This work, neon matrix

Table S3.3. Vibrational frequencies computed for octahedral PtF_6 at the 2c-X2C DFT level.

2c-X2C	PtF_6 intensity (%)	PtF_6 frequency in cm^{-1}
B3LYP	0	222
	0	223
	0	224
	0	253
	0	254
	0	256
	8	274
	8	276
	8	276
	0	608
	0	613
	0	669
	100	697
	100	697
	100	698
PBE0	0	229
	0	230
	0	231
	0	261
	0	262
	0	263
	8	282
	8	283
	8	284
	0	632
	0	638

	0	700
	100	724
	100	725
	100	725
	0	222
	0	223
	0	224
	0	249
	0	250
	0	251
	8	275
TPSSh	8	276
	8	276
	0	613
	0	619
	0	669
	100	698
	100	698
	100	699

References to Part 3

[26] J. Holloway, G. Stanger, E. G. Hope, W. Levason, J. S. Ogden, *J. Chem. Soc. Dalton Trans.* **1988**, 1341–1345.

[27] K. Seppelt, *Chem. Rev.* **2015**, *115*, 1296–1306.

[28] a) L. Alvarez-Thon, J. David, R. Arratia-Pérez, K. Seppelt, *Phy. Rev. A* **2008**, *77*, 034502; b) J. David, P. Fuentealba, A. Restropo, *Chem. Phys. Lett*. **2008**, *457*, 42–44.

[29] a) A. D. Richardson, K. Hedberg, G. M. Lucier, *Inorg. Chem.* **2000**, *39*, 2787 –2793; b) R. Marx, K. Seppelt, R. M. Ibbertson, *J. Chem. Phys.* **1996**, *104*, 7658–7664.

Table S3.4. Low-energy electronic excitation wavelengths of PtF_6 computed using linear-response TDDFT at different two-component levels**.**

Functional	Hamiltonian	$\Delta\lambda$[a)] in nm	oscillator strength in a.u.
B3LYP	2c-X2C	429.8<br>341.3	0.0164<br>0.0173
TPSSh	2c-X2C	433.8<br>345.7	0.0186<br>0.0175
PBE0	2c-X2C	403.0<br>320.3	0.0169<br>0.0206
B3LYP	2c-ZORA[c)]	429.7<br>(–425.9)<br>342.6<br>(–340.4)	0.0173<br>(0.0184)<br>0.0172<br>(0.018)
PBE0	2c-ZORA	406.8<br>(–404)<br>324.5<br>(–322.8)	0.0177<br>(–0.0184)<br>0.02<br>(0.0201)
TPSSh	2c-ZORA	-[b)]<br>(417.6)<br>-[b)]<br>(336.6)	-<br>(0.0209)<br>-<br>(0.0185)
Exp.		436.3 [d)]<br>311.7 [d)]	

a) Excitations are threefold degenerate. Values in parentheses were obtained with the ALDA approximation.
b) No full kernel available for TPSSh.
c) For 2c-ZORA (but not for 2c-X2C) one negative excitation energy (threefold degenerate) is observed. This reflects an instability with respect to a lower-lying triplet state, which appears to be absent for the X2C computations with Turbomole (as well as in LDA-based computations in ADF).
d) This work, neon matrix.

Part 4: Structures, vibrational frequencies and electronic transitions of PtF_4.

Table S4.1 Electronic transitions (λ in nm) and their vibrational spacing ($\Delta\tilde{\nu}$ in cm^{-1}) obtained for molecular PtF_4 in a solid neon matrix. Absorbance maxima are indicated by bold values.

λ [nm] [a)]	$\Delta\tilde{\nu}$ [cm^{-1}]	Mean vibrational spacing $\Delta\tilde{\nu}_{av}$ [cm^{-1}]
249.8		542 cm^{-1}
253.7	615.4	
256.6	445.4	
260.2	539.2	
263.8	524.5	
267.6	538.3	
**271.5 (36832.4 cm^{-1})**	536.8	
275.7	561.1	
279.8	531.5	
284.2	553.3	
288.5	524.5	
293.5	590.4	
**325.0 (30769.2 cm^{-1})**		Not resolved
494.1 vw (20238.8 cm^{-1})		
513.5 vw (19474.2 cm^{-1})		

[a)]: vw: very weak.

Computational results on PtF_4

We have evaluated the lowest-energy singlet and triplet configurations of PtF_4 at 1c-X2C and 2c-X2C levels. In all cases, the triplet is slightly below the singlet (see Table S4.2). Table S4.3 shows structural details and vibrational frequencies of the D_{2h} symmetrical triplet state. Most notably, the 2c-computations provide only very small distortions away from a regular D_{4h} structure, while the angles deviate a bit more from 90° at 1c-levels. This is also reflected in the splitting between the two Pt–F stretching frequencies, which is about 13–14 cm^{-1} at 1c-levels (about 20 cm^{-1} at 1c-ECP CCSD(T) level) but only 4–5 cm^{-1} at 2c-levels. Differences between the different quasirelativistic Hamiltonians (X2C vs. ZORA) or codes (Turbomole vs. ADF) are much smaller than this. The splitting at 2c-level agrees well with the observed splitting of 4 cm^{-1}. Here B3LYP frequencies are somewhat too low, TPSSh frequencies less so, while PBE0 frequencies are too high by similar amounts. If we assume the experimental bands at 712 cm^{-1} and 716 cm^{-1} to belong to PtF_4, the computed stretching frequencies should be shifted up by 6 cm^{-1} and 12 cm^{-1}, respectively, compared to PtF_6 (cf. Tables S3.2, S3.3). The TPSSh 2c-X2C results give shifts of 4 cm^{-1} and 9 cm^{-1}, not too far away from the observations. The up-frequency shifts with the other functionals are somewhat smaller, as are the ZORA results. For example, the PBE0 2c-X2C results would suggest that only one of the two frequencies in PtF_4 is above that of PtF_6. Such small frequency shifts are obviously at the limit of a theory-experiment comparison given

that the experimental data also depend on the matrix. We conclude that the IR data for PtF_4 are consistent with the spectra observed for the new species, and that SO coupling reduces the splitting between stretching modes.

Table S4.2. Relative energies (also with zero point vibrational energy correction) of the lowest-energy singlet ($^1A_{1g}$, D_{4h}) and triplet ($^3B_{2g}$, D_{2h}) configurations of PtF_4 at 1c-X2C and 2c-X2C levels.

functional	Level	ΔE	$\Delta(E+\text{ZPEV})$
		in kJ/mol	
B3LYP	1c-X2C	–57.74	-59.70
	2c-X2C	–10.83	-11.14
PBE0	1c-X2C	–62.09	-66.31
	2c-X2C	–13.96	-14.45
TPSSh	1c-X2C	–55.90	-57.91
	2c-X2C	–10.95	-11.52

Table S4.3. One- and two-component X2C and ZORA DFT computations of structures and vibrational frequencies of the lowest triplet state of PtF_4 ($^3B_{2g}$, D_{2h}).

Functional	Level	d(Pt–F) in Å	α in deg	ν_1 (b_{2u}) in cm^{-1}	ν_2 (b_{3u}) in cm^{-1}
B3LYP	2c-X2C	1.857	90.69	698	701
	1c-X2C	1.853	92.36	696	708
	2c-ZORA	1.86	90.83	699	703
PBE0	2c-X2C	1.839	90.84	721	725
	1c-X2C	1.835	92.37	720	733
	2c-ZORA	1.841	91.00	724	728
TPSSh	2c-X2C	1.852	91.06	702	707
	1c-X2C	1.85	92.40	701	712
	2c-ZORA	1.855	91.10	704	709
CCSD(T)	1c-ECP [a)]	1.834	92.66	710	730
Exp.[b)]				713	717

a) With aug-cc-pVTZ(-PP) basis.

b) This work, neon matrix.

Table S4.4. One- and two-component X2C and ZORA DFT computations of structures and vibrational frequencies of the lowest singlet state of PtF_4 ($^1A_{1g}$,D_{4h}, calculated in D_{2h} symmetry)

Functional	Level	d(Pt–F) in Å	ν_1 (b_{2u}) in cm^{-1}	ν_1 (b_{3u}) in cm^{-1}
B3-LYP	2c-X2C	1.853	702	703
	1c-X2C	1.844	707	707
PBE0	2c-X2C	1.835	726	726
	1c-X2c	1.827	725	725
TPSSh	2c-X2C	1.849	707	709
	1c-X2C	1.842	705	705

Table S4.5. Vibrational frequencies computed for planar triplet PtF_4 ($^3B_{2g}$, D_{2h}) at different 1c-X2C and 2c-X2C levels.

1c-X2C	PtF_4 ($^3B_{2g}$, D_{2h})		
	intensity (%)	symmetry	frequency in cm^{-1}
B3LYP	0	A_u	161
	8	B_{2u}	221
	6	B_{1u}	232
	5	B_{3u}	255
	0	A_g	275
	0	B_{1g}	355
	0	A_g	682
	100	B_{2u}	696
	93	B_{3u}	708
PBE0	0	A_u	165
	8	B_{2u}	227
	6	B_{1u}	238
	4	B_{3u}	261
	0	A_g	281
	0	A_g	711
	100	B_{2u}	720
	93	B_{3u}	733
TPSSh	0	A_u	165
	8	B_{2u}	223
	6	B_{1u}	234
	5	B_{3u}	256
	0	A_g	272
	0	B_{1g}	426
	0	A_g	685
	100	B_{2u}	701
	93	B_{3u}	712

2c-X2C	PtF_4 ($^3B_{2g}$, D_{2h})	
	intensity (%)	Frequency in cm^{-1}
B3LYP	0	154
	8	214
	5	242
	4	251
	0	256
	0	570
	0	670
	100	698
	98	701
PBE0	0	161
	8	220
	4	250

	0	258
	4	260
	0	562
	0	697
	100	721
	98	725
TPSSh	0	161
	7	223
	5	238
	4	252
	0	255
	0	579
	0	675
	100	702
	97	707

Table S4.6. Vibrational frequencies computed for planar singlet PtF_4 ($^1A_{1g}$, D_{4h}) at different 1c-X2C and 2c-X2C levels.

1c-X2C	PtF_4 ($^1A_{1g}$, D_{4h})		
	intensity (%)	symmetry	frequency in cm^{-1}
B3LYP	13	B_{3u}	196
	13	B_{2u}	196
	0	A_u	219
	3	B_{1u}	288
	0	A_g	302
	0	B_{1g}	619
	0	A_g	690
	100	B_{3u}	701
	100	B_{2u}	701
PBE0	13	A_u	198
	13	B_{2u}	198
	0	B_{3u}	227
	3	A_g	297
	0	B_{1u}	310
	0	B_{1g}	642
	0	A_g	719
	100	B_{2u}	725
	100	B_{3u}	725
TPSSh	13	B_{3u}	215
	12	B_{2u}	216
	0	A_u	233
	0	A_g	305
	6	B_{1u}	311
	0	B_{1g}	629
	0	A_g	692
	100	B_{2u}	705
	100	B_{3u}	705

2c-X2C	PtF_4 ($^1A_{1g}$, D_{4h})	
	intensity (%)	frequency in cm^{-1}
B3LYP	0	170
	7	228
	6	239
	6	239
	0	261
	0	588
	0	678
	100	702
	100	703
PBE0	0	177
	6	236
	5	244
	5	245
	0	269
	0	609
	0	707
	100	726
	100	726
TPSSh	0	176
	6	233
	6	239
	6	240
	0	260
	0	599
	0	681
	100	706
	100	707

Table S4.7. Comparison of different approximations for TDDFT computations of low-energy electronic excitation wavelengths of triplet PtF_4 ($^3B_{2g}$, D_{2h}) using the B3LYP functional.

approximations	$\Delta\lambda$ in nm	oscillator strength in a.u.
2c-ZORA[a)]	327.3	0.0268
	323.5	0.0196
ALDA	280.9	0.0283
	280.7	0.0134
TDA	271.5	0.0230
	271.1	0.0430
2c-ZORA[a)]	330.6	0.0265
	326.8	0.0190
full kernel	282.3	0.0223
	281.9	0.0123
TDA	272.6	0.0227
	272.3	0.0416
1c-ZORA	323.0	0.0495
	308.7	0.0321
ALDA	284.9	0.0480
TDA	277.5	0.0596
1c-ZORA	339.9	0.0506
	319.3	0.0408
ALDA	293.8	0.0194
TDDFT	287.4	0.0274

a) 2c-ZORA is possible only within the TDA.

Table S4.8. 2c-ZORA TDDFT results of excitations ($\Delta\lambda$) for triplet PtF_4 ($^3B_{2g}$, D_{2h}) using the TDA for different functionals.

approximations		$\Delta\lambda$ in nm	oscillator strength in a.u.
B3LYP	ALDA	327.3	0.0268
		323.5	0.0196
		280.9	0.0283
		280.7	0.0134
		271.5	0.0230
		271.1	0.0430
	full kernel	330.6	0.0265
		326.8	0.0190
		282.3	0.0223
		281.9	0.0123
		272.6	0.0227
		272.3	0.0416
PBE0	ALDA	365.2	0.0117
		307.5	0.0296
		303.8	0.0200
		272.6	0.0128
		261.7	0.0284
		260.1	0.0116
		253.9	0.0168
		253.5	0.0379
	full kernel	366.6	0.0117
		310.2	0.0290
		306.4	0.0195
		273.9	0.0122
		262.6	0.0253
		261.2	0.0108
		254.9	0.0180
		254.4	0.0377
TPSSh	ALDA[a)]	319.9	0.0155
		288.7	0.0168
		288.4	0.0256
		274.0	0.0488
		270.5	0.0662

a) in case of TPSSh only the ALDA kernel is available

Table S4.9. 2c-X2C TDDFT results of excitations ($\Delta\lambda$) for singlet PtF_4 ($^1A_{1g}$, D_{4h}) using different functionals.

2c-X2C	$\Delta\lambda$ [a)] in nm	oscillator strength in a.u.
B3LYP	301.5	0.01551
	301.5	0.01551
	283.5	0.04555
	283.5	0.04555
PBE0	283.0	0.01623
	283.0	0.01623
	267.3	0.05000
	267.3	0.05001
TPSSh	313.5	0.01521
	313.5	0.01521
	288.4	0.05161
	288.4	0.05161

a) TD-DFT excitation for singlet PtF_4 (using ADF) shows that degeneracy comes from E-type symmetry.

Part 5: Computed structures and vibrational spectra of PtF_5 ($^2B_2$, C_{4v})

Table S5.1. Structure and vibrational frequencies for $^2B_2$-PtF_5 at 1c-X2C and 2c-X2C DFT and 1c-ECP CCSD(T) levels

		α in deg	β in deg	d(Pt–F)$_{eq}$ in Å	d(Pt–F)$_{ax}$ in Å	$\nu_{as}(PtF_4)$ in cm^{-1}
B3LYP	1c-X2C	92.26	89.93	1.872	1.860	682
	2c-X2C	93.22	89.85	1.875	1.862	680
PBE0	1c-X2C	92.08	89.92	1.853	1.839	706
	2c-X2C	92.93	89.88	1.856	1.840	705
TPSSh	1c-X2C	92.20	89.92	1.870	1.855	685
	2c-X2C	92.96	89.85	1.872	1.857	684
CCSD(T)	1c-ECP	91.93	89.94	1.854	1.856	705
Exp.[a)]						691

[a)]: This work: in solid neon matrix

Table S5.2. Vibrational frequencies computed for molecular PtF_5 ($^2B_2$, C_{4v}) at different 1c-X2C and 2c-X2C levels.

1c-X2C	PtF_5 ($^2B_2$, C_{4v})		
	intensity (%)	Symmetry [a)]	frequency in cm^{-1}
B3LYP	0	B_1	107
	0	B_2	107
	0	A_2	203
	9	B_1	231
	9	B_2	231
	0	A_1	262
	7	A_1	264
	0	A_2	621
	0	A_1	664
	100	B_1	682
	100	B_2	682
	3	A_1	690
PBE0	0	B_2	116
	0	B_1	116
	0	A_2	214
	9	B_2	236
	9	B_1	236
	0	A_1	269
	6	A_1	275
	0	A_2	645
	1	A_1	694
	100	B_1	706
	100	B_2	706
	3	A_1	721
TPSSh	1	B_1	96
	1	B_2	96
	0	A_2	204
	9	B_1	234
	9	B_2	234
	0	A_1	259
	7	A_1	264
	0	A_2	629
	0	A_1	665
	100	B_1	685
	100	B_2	685
	4	A_1	697

[a)]: Calculated in C_{2v} symmetry

2c-X2C	PtF_5 ($^2B_2$, C_{4v})	
	intensity (%)	frequency in cm^{-1}
B3LYP	1	129
	1	131
	0	178
	10	236
	6	244
	6	245
	0	247
	0	608
	11	637
	1	665
	100	680
	100	680
PBE0	1	139
	1	140
	0	189
	9	247
	6	250
	6	251
	0	255
	0	633
	9	670
	3	696
	100	705
	99	705
TPSSh	1	123
	1	125
	0	183
	10	239
	6	244
	5	244
	2	245
	0	619
	9	646
	3	670
	100	684
	100	684

Part 6: Computed structures and vibrational frequencies of difluorine complexes $PtF_n \cdot F_2$, n = 4, 5.

Due to the confinement in the solid matrix, it cannot be excluded that photolysis and F_2 elimination from PtF_6 yields a $PtF_4 \cdot F_2$ complex or at least a close proximity of F_2 to PtF_4. To obtain insight into the stability and spectral signatures of such complexes we have computed $PtF_4 \cdot F_2$ complexes in both side-on and end-on coordination to platinum (see Figure 7, main text), again comparing the lowest triplet and singlet states. As the interactions turned out to be weak, we included dispersion interactions in the 1c- and 2c-DFT computations by adding either atom-additive corrections (Grimme's D3 and D4 corrections) or in one case a proper VV10 van-der-Waals functional (in 1c-computations, using the ωB97M-V functional in Turbomole). The interaction energies are generally small (Table S6.1), consistent with a weak van-der-Waals complex. Only the VV10-based ωB97M-V results give a somewhat larger binding, but still in the range of a van-der-Waals complex. The dominance of dispersion corrections is demonstrated by giving also the pure D3 contribution to the B3LYP-D3 binding energy (Table S6.1): it is almost identical to the total binding energy. These observations hold for both the side-on and the end-on configuration. The former is somewhat lower in energy at all levels. However, the energy differences are so small that a fluxional nature of the complex may be assumed, provided the matrix environment permits the motion.

Table S6.1. Computed $PtF_4 \cdot F_2$ binding energies in kJ/mol at different dispersion-corrected one- and two-component X2C DFT levels (triplet- and singlet-state results).

	side-on		end-on	
triplet state	1c-X2C	2c-X2C	1c-X2C	2c-X2C
B3LYP-D3	6.95	7.24	3.60	3.51
PBE0-D3	6.32	6.57	3.01	2.97
TPSSh-D3	5.94	6.11	3.22	3.14
ωB97M-V	11.25	-	5.27	-
B3LYP-D4	5.56	5.90	2.72	2.64
B3LYP-D3(BJ)	5.24	5.58	2.50	2.41
D3[a)] (B3LYP)	6.99	7.11	4.60	4.60
singlet state	1c-X2C	2c-X2C	1c-X2C	2c-X2C
B3LYP-D3	6.36	6.94	4.32	3.71
PBE0-D3	5.76	6.28	3.57	3.13
TPSSh-D3	5.41	5.89	4.01	3.35
ωB97M-V	10.31	-	5.76	-
B3LYP-D4	5.08	5.55	3.50	2.85
B3LYP-D3(BJ)	4.78	5.24	3.25	2.61
D3[a)] (B3LYP)	6.82	6.98	4.56	4.60

[a)]: Isolated dispersion contribution to B3LYP-D3 result.

Tables S6.2 - S6.5 provide computed structural and vibrational data for the side-on and end-on $PtF_4{\cdot}F_2$ complexes, respectively, at various 1c- and 2c-levels. The F–F distances are generally very close to those of free F_2, and the long Pt–F_2 distances also corroborate the relatively weak interactions. The same holds for the internal structures of the PtF_4 fragment, which are close to the free PtF_4 results at the given computational level and also exhibit the abovementioned differences between 1c- and 2c-optimizations. Consequently, the vibrational frequencies are altered very little compared to free PtF_4. Let us pick the 2c-X2C results with the TPSSh functional as an example (with D3 corrections for the complex). Changes compared to free PtF_4 ($^3B_2$) are ca. 3 cm^{-1} for the higher of the two frequencies, while the lower one remains almost unchanged (below 1 cm^{-1}). The same holds for the other computational levels. These small modifications render an experimental identification of the $PtF_4{\cdot}F_2$ interactions difficult.

Table S6.2. Structures and vibrational frequencies computed for side-on triplet and singlet $PtF_4{\cdot}F_2$ at 1c-X2C and 2c-X2C levels.

triplet		α in deg	d(Pt–F) in Å	d(F–F) in Å	d(Pt...F) in Å	ν_1 in cm^{-1}	ν_2 in cm^{-1}
B3LYP	2c-X2C	90.7	1.857	1.397	3.728	698	700
B3LYP-D3	1c-X2C	92.3	1.853	1.398	3.261	703	716
	2c-X2C	90.7	1.857	1.398	3.245	701	703
PBE0	2c-X2C	90.8	1.839	1.376	3.497	724	726
PBE0-D3	1c-X2C	92.4	1.836	1.377	3.289	727	740
	2c-X2C	90.8	1.840	1.377	3.270	724	727
TPSSh	2c-X2C	91.0	1.852	1.400	3.831	704	707
TPSSh-D3	1c-X2C	92.4	1.850	1.401	3.371	707	720
	2c-X2C	91.0	1.852	1.401	3.728	705	708
ωB97M-V [a)]	1c-X2C	92.3	1.837	1.383	3.261	726	738

singlet		α in deg	d(Pt–F) in Å	d(F–F) in Å	d(Pt...F) in Å	ν_1 in cm^{-1}	ν_2 in cm^{-1}
B3LYP	2c-X2C	90.0	1.853	1.398	3.777	703	705
B3LYP-D3	1c-X2C	90.0	1.845	1.398	3.287	701	701
	2c-X2C	90.0	1.853	1.398	3.264	704	705
PBE0	2c-X2C	90.0	1.835	1.377	3.540	728	729
PBE0-D3	1c-X2C	90.0	1.827	1.377	3.326	724	724
	2c-X2C	90.0	1.835	1.377	3.295	728	729
TPSSh	2c-X2C	90.0	1.849	1.400	3.868	708	709
TPSSh-D3	1c-X2C	90.0	1.842	1.400	3.417	705	705
	2c-X2C	90.0	1.849	1.401	3.379	708	708
ωB97M-V [a)]	1c-X2C	90.0	1.827	1.383	3.225	729	730

[a)]:Use of the range-separated ωB97M-V with VV10 nonlocal correlation is possible only at 1c level.

Table S6.3. Structures and vibrational frequencies computed for end-on $PtF_4 \cdot F_2$ at 1c-X2C and 2c-X2C

triplet		α in deg	d(Pt–F) in Å	d(F–F) in Å	d(Pt...F) in Å	ν_1 in cm^{-1}	ν_2 in cm^{-1}
B3LYP	2c-X2C	90.71	1.857	1.397	7.619	700	702
B3LYP-D3	1c-X2C	92.35	1.852	1.398	3.169	704	717
	2c-X2C	90.69	1.856	1.398	3.169	702	704
PBE0	2c-X2C	90.83	1.839	1.375	3.565	723	726
PBE0-D3	1c-X2C	92.38	1.835	1.375	3.213	728	741
	2c-X2C	90.83	1.839	1.376	3.207	726	728
TPSSh	2c-X2C	91.09	1.850	1.399	3.850	704	708
TPSSh-D3	1c-X2C	92.38	1.849	1.401	3.220	702	713
	2c-X2C	91.05	1.851	1.401	3.219	705	709
ωB97M-V [a)]	1c-X2C	92.38	1.836	1.381	3.151	730	742

singlet		α in deg	d(Pt–F) in Å	d(F–F) in Å	d(Pt...F) in Å	ν_1 in cm^{-1}	ν_2 in cm^{-1}
B3LYP [b)]	2c-X2C	90.0	1.853	1.398	3.590	704	705
B3LYP-D3	1c-X2C	90.0	1.844	1.399	3.127	702	702
	2c-X2C	90.0	1.853	1.398	3.159	705	706
PBE0 [b)]	2c-X2C	90.0	1.835	1.377	3.465	728	729
PBE0-D3	1c-X2C	90.0	1.827	1.377	3.175	725	725
	2c-X2C	90.0	1.835	1.376	3.201	729	730
TPSSh[b)]	2c-X2C	90.0	1.849	1.400	3.861	708	709
TPSSh-D3	1c-X2C	90.0	1.841	1.403	3.134	706	706
	2c-X2C	90.0	1.849	1.401	3.199	709	709
ωB97M-V [a)]	1c-X2C	90.0	1.827	1.382	3.124	730	731

[a)]: Use of the range-separated ωB97M-V with VV10 nonlocal correlation is possible only at 1c-X2C level.

[b)]: For functionals without dispersion correction C_{4v} symmetry was broken during geometry optimization.

Table S6.4. Vibrational frequencies computed for a molecular side-on triplet $PtF_4{\cdot}F_2$ complex (C_{2v}) at different 1c-X2C and 2c-X2C levels.

1c-X2C	$PtF_4{\cdot}F_2$ (side-on)		
	intensity (%)	symmetry	frequency in cm^{-1}
B3LYP-D3	0	A_1	63
	0	B_2	143
	0	B_1	147
	0	A_2	253
	5	B_2	295
	0	A_2	296
	0	B_1	298
	8	A_1	307
	0	A_1	310
	3	B_1	323
	0	A_2	629
	0	A_1	687
	100	B_2	703
	92	B_1	716
	0	A_1	1072
PBE0-D3	0	A_1	53
	0	B_2	140
	0	B_1	144
	0	A_2	255
	0	A_2	274
	0	B_1	279
	5	B_2	301
	8	A_1	310
	0	A_1	316
	3	B_1	329
	0	A_2	654
	0	A_1	715
	100	B_2	727
	92	B_1	740
	0	A_1	1120
TPSSh-D3	0	A_1	43
	0	B_2	146
	0	B_1	149
	0	A_2	258
	0	A_2	280
	0	B_1	281
	5	B_2	296

	0	A_1	305
	7	A_1	310
	3	B_1	323
	0	A_2	635
	0	A_1	689
	100	B_2	707
	91	B_1	720
	0	A_1	1068

2c-X2C	$PtF_4{\cdot}F_2$ (side-on)	
	intensity (%)	frequency in cm^{-1}
B3LYP-D3	0	60
	0	62
	0	69
	0	131
	0	138
	0	182
	10	237
	4	261
	3	269
	0	270
	0	621
	0	674
	100	701
	100	703
	0	1046
PBE0-D3	0	52
	0	67
	0	74
	0	130
	0	148
	0	189
	9	245
	3	270
	3	277
	0	278
	0	646
	0	702
	100	724
	97	727
	0	1097

	0	40
	0	44
	0	54
	0	80
	0	87
	0	176
	9	233
TPSSh-D3	5	251
	3	264
	0	265
	0	614
	0	678
	98	705
	100	708
	0	1042

Table S6.5. Vibrational frequencies computed for a molecular end-on triplet $PtF_4{\cdot}F_2$ complex (C_{2v}) at different 1c-X2C and 2c-X2C levels.

1c-X2C	$PtF_4{\cdot}F_2$ (end-on)		
	intensity (%)	symmetry	frequency in cm^{-1}
	0	A_1	55
	0	B_2	149
	0	B_1	153
	0	A_2	254
	0	B_2	276
	0	B_1	277
	5	B_2	295
B3LYP-D3	8	A_1	308
	0	A_1	309
	3	B_1	323
	0	A_2	630
	0	A_1	688
	100	B_2	704
	94	B_1	717
	0	A_1	1059
	0	A_1	45
	0	B_2	145
	0	B_1	150
	0	A_2	255
PBE0-D3	0	B_1	274
	0	B_2	274
	5	B_2	301
	7	A_1	311

	0	A_1	316
	3	B_1	330
	0	A_2	655
	0	A_1	716
	100	B_2	728
	94	B_1	741
	0	A_1	1118
TPSSh-D3	0	B_2	21
	0	B_1	25
	0	A_1	39
	0	B_1	58
	0	B_2	62
	0	A_2	168
	8	B_2	225
	6	A_1	236
	5	B_1	258
	0	A_1	275
	0	A_2	433
	0	A_1	686
	100	B_2	702
	93	B_1	713
	3	A_1	1029

2c-X2C	$PtF_4{\cdot}F_2$ (end-on)	
	intensity (%)	frequency in cm^{-1}
B3LYP-D3	0	49
	0	60
	0	66
	0	112
	0	127
	0	179
	9	236
	4	261
	3	269
	0	271
	0	620
	0	676
	98	702
	100	704
	1	1038
PBE0-D3	0	41
	0	71
	0	75
	0	129

	0	144
	0	189
	8	246
	3	270
	3	277
	0	278
	0	648
	0	703
	98	726
	100	728
	0	1095
TPSSh-D3	0	37
	0	43
	0	52
	0	70
	0	79
	0	177
	8	234
	4	251
	3	265
	0	266
	0	615
	0	679
	96	705
	100	709
	3	1030

[a]: Calculated in C_1 symmetry

We also considered complexes of PtF_5 with additional F_2. In contrast to PtF_4, here an end-on bent C_s-symmetrical coordination with a Pt–F–F angle of around 112°–115° (Figure 7 main text) has been found to be energetically preferable over a C_{2v}-symmetrical side-on coordination (by about 37 kJ/mol at B3LYP 1c-X2C level). Binding energies, structure and vibrational frequencies for doublet $PtF_5{\cdot}F_2$ (C_s end-on) complexes are given in Tables S6.6 S6.7 and S6.8. In contrast to $PtF_4{\cdot}F_2$, the larger interaction energy is not anymore dominated by dispersion interactions. Indeed, the interaction seems so strong at TPSSh-D3 level that the F–F bond dissociates during structure optimization.

Table S6.6. Computed $PtF_5 \cdot F_2$ binding energies in kJ/mol at different dispersion-corrected one- and two-component X2C DFT levels.

functional/dispersion	C_s	
	1c-X2C	2c-X2C
B3LYP-D3	39.73	34.95
PBE0-D3	41.03	36.56
TPSSh-D3[a)]	-	-
ωB97M-V	55.90	-
B3LYP-D4	40.13	35.47
B3LYP-D3(BJ)	39.97	35.09
D3 [b)] (B3LYP)	7.70	7.73

[a)]: F–F bond dissociation found during optimization.
[b)]: Isolated dispersion contribution to B3LYP-D3 result.

Table S6.7. Structure and vibrational frequencies for doublet $PtF_5 \cdot F_2$ (C_s end-on) at various computational levels.

		α in deg	d(Pt–F)$_{ax}$ in Å	d(Pt–F) in Å	d(F–F) in Å	ν_1 (a') in cm^{-1}	ν_2 (a'') in cm^{-1}	ν_3 (a') in cm^{-1}
B3LYP-D3	1c-X2C	113.42	1.861	2.234	1.407	679	679	691
	2c-X2C	112.73	1.856	2.281	1.403	676	676	677
PBE0-D3	1c-X2C	112.78	1.842	2.212	1.383	703	703	719
	2c-X2C	112.42	1.836	2.249	1.381	700	700	705
TPSSh-D3[a)]	1c-X2C	-	-	-	-	-	-	-
	2c-X2C	-	-	-	-	-	-	-

[a)]: F–F bond dissociation found during optimization.

Table S6.8. Vibrational frequencies computed for a molecular end-on doublet $PtF_5{\cdot}F_2$ complex (C_s) at different 1c-X2C and 2c-X2C levels.

1c-X2C	$PtF_5{\cdot}F_2$ (C_s end-on)		
	intensity (%)	symmetry	frequency in cm^{-1}
B3LYP-D3	0	A''	60
	0	A'	86
	0	A''	117
	1	A'	157
	0	A'	177
	0	A''	186
	0	A''	224
	10	A'	232
	9	A''	236
	0	A'	265
	5	A'	270
	4	A'	278
	0	A''	615
	0	A'	660
	100	A''	679
	95	A'	679
	16	A'	691
	5	A'	962
PBE0-D3	0	A''	56
	0	A'	92
	0	A''	128
	1	A'	161
	1	A'	187
	0	A''	193
	0	A''	233
	9	A'	238
	8	A''	241
	0	A'	271
	1	A'	285
	10	A'	288
	0	A''	638
	0	A'	689
	100	A''	703
	96	A'	703
	15	A'	719
	1	A'	1037

2c-X2C	$PtF_5 \cdot F_2$ (C_s end-on)	
	intensity (%)	frequency in cm^{-1}
B3LYP-D3	0	82
	0	106
	2	146
	1	182
	0	188
	0	202
	7	238
	6	247
	0	249
	13	252
	1	257
	0	604
	6	646
	47	676
	100	676
	68	677
	1	998
PBE0-D3	0	16
	0	88
	0	114
	1	151
	1	192
	0	196
	0	213
	6	248
	6	253
	0	255
	10	264
	5	269
	0	627
	6	674
	100	700
	96	700
	16	705
	1	1058

Part 7: Computed structures and vibrational spectra of PtF_n, n = 1–3.

To assist in the characterization of additional species found in laser ablation experiments, computations of the lower platinum fluorides at various computational levels have been carried out. Table S7.1 provides results for PtF in its doublet state. Agreement between computed and experimental Pt–F stretching frequencies is comparable as for the higher fluorides (see above), with the ECP-based CCSD(T) value being slightly too low and PBE0 again providing the highest value. The slight lowering of the stretching frequency in going from 1c- to 2c-levels reflects SO effects. Transfer to the 1c-ECP CCSD(T) value would enhance somewhat the underestimate found at coupled-cluster level. Interestingly, at the same time SO coupling shortens the Pt–F bond somewhat, in a nonintuitive way.

The higher stretching frequency of linear PtF_2 in its triplet ground state (Table S7.2) reflects the bond strengthening achieved by improved sd-hybridization compared to the monohydride (see higher 6s population in Table S7.6). The lower stretching frequency combined with a lengthening of the bond from the 1c-X2C to 2c-X2C levels reflects the SO effects. Transferring this SO-induced lowering of the stretching frequency to the otherwise too high 1c-ECP CCSD(T) result would now bring also the coupled-cluster data into good agreement with experiment. The relative behavior of the different DFT functionals follows the observations made for the other platinum fluorides (see above).

Table S7.1. Computed bond length and vibrational frequency for PtF ($^2\Sigma^+$) at 1c-X2C/2c-X2C DFT and 1c-ECP CCSD(T) levels

PtF ($^2\Sigma^+$)		d(Pt–F) in Å	ν_1 (Σ^+) in cm^{-1}
B3LYP	1c-X2C	1.895	600
	2c-X2C [b]	1.889 (1.873)	602 (594)
PBE0	1c-X2C	1.878	616
	2c-X2C [b]	1.874 (1.859)	619 (607)
TPSSh	1c-X2C	1.882	618
	2c-X2C [b]	1.877 (1.877)	620 (609)
CCSD(T)	1c-ECP	1.892	595
Exp.[a]			605

a) This work, neon matrix
b) Data for z-direction of spin magnetization. In parenthesis data for x/y-direction of spin magnetization. The x/y-direction has lower energy than solution with magnetization in z direction, but total spin magnetization is close to zero what suggests broken symmetry solution.

Table S7.2. Computed bond length and vibrational frequencies for the linear triplet ground state of PtF_2 ($^3\Sigma_g^-$) at 1c-X2C/2c-X2C DFT and 1c-ECP CCSD(T) levels.

PtF_2 ($^3\Sigma_g^-$)		d(Pt–F) in Å	ν_1 (Σ_g^+) in cm^{-1}
B3LYP	1c-X2C	1.833	725
	2c-X2C	1.849	705
PBE0	1c-X2C	1.818	744
	2c-X2C	1.833	723
TPSSh	1c-X2C	1.823	735

	2c-X2C	1.837	710
CCSD(T)	1c-ECP	1.830	731
Exp. [a]			710

[a] This work, neon matrix.

Table S7.3. Computed vibrational frequencies for the linear triplet ground state of PtF_2 ($^3\Sigma_g^-$) at different 1c-X2C/2c-X2C DFT levels.

1c-X2C	PtF$_2$ ($^3\Sigma_g^-$) intensity (%)	symmetry [a)]	frequency in cm^{-1}
B3LYP	3	B_{3u}	166
	3	B_{2u}	166
	0	A_g	669
	100	B_{1u}	725
PBE0	3	B_3	167
	3	B_{2u}	167
	0	A_g	688
	100	B_{1u}	744
TPSSh	2	B_{3u}	168
	2	B_{2u}	168
	0	A_g	678
	100	B_{1u}	735

[a)]: Calculated in D_{2h} symmetry.

2c-X2C	PtF$_2$ ($^3\Sigma_g^-$) intensity (%)	frequency in cm^{-1}
B3LYP	3	169
	3	171
	0	636
	100	705
PBE0	3	172
	0	653
	100	723
TPSSh	3	172
	3	173
	0	645
	100	710

Table S7.4. Computed structural data and vibrational frequencies for $^2A_2$ C_{2v} PtF_3 at 1c-X2C/2c-X2C DFT and 1c-ECP CCSD(T) levels

PtF_3		α(F1–Pt–F3) in deg	d(Pt–F1/3)	d(Pt–F2)	ν_1 (A_1) in cm^{-1}	ν_1 (B_1) in cm^{-1}
B3LYP	1c-X2C	175.51	1.884	1.865	634	683
	2c-X2C	171.13	1.883	1.871	620	679
PBE0	1c-X2C	175.82	1.865	1.848	658	705
	2c-X2C	171.81	1.863	1.854	630	696
TPSSh	1c-X2C	175.80	1.876	1.860	645	690
	2c-X2C	172.26	1.873	1.865	624	684
CCSD(T)	1c-ECP	175.82	1.875	1.854	644	702

Table S7.5. Computed vibrational frequencies for the triplet ground state of PtF_3 ($^2A_2$, C_{2v}) at different 1c-X2C/2c-X2C DFT levels.

1c-X2C	PtF_3 ($^2A_2$, C_{2v}) intensity (%)	Symmetry [a]	frequency in cm^{-1}
B3LYP	6	B_2	129
	2	B_1	130
	3	A_1	243
	16	A_1	634
	0	A_1	669
	100	B_1	683
PBE0	5	B_2	135
	2	B_1	140
	3	A_1	252
	16	A_1	658
	0	A_1	694
	100	B_1	705
TPSSh	5	B_2	125
	2	B_1	126
	3	A_1	244
	16	A_1	645
	0	A_1	674
	100	B_1	690

2c-X2C	PtF_3 ($^2A_2$, C_{2v}) intensity (%)	frequency in cm^{-1}
B3LYP	3	174
	5	206
	4	222
	19	620.
	0	660
	100	679

PBE0	2	153
	5	188
	5	211
	22	630
	1	683
	100	696
TPSSh	2	150
	5	189
	5	211
	21	624
	1	666
	100	684

[a]: Calculated in C_1 symmetry.

Table S7.6. Natural (NPA) charges and natural electron configurations for PtF_n (n =1–6) series.

B3LYP 2c-X2C	Q(Pt)	Q(F)
PtF	0.585	–0.585
PtF_2	1.049	–0.525
PtF_3	1.529	–0.531 (x2), –0.467 (x1)
PtF_4	1.826	–0.456
PtF_5	2.183	–0.461 (x4), –0.338 (x1)
PtF_6	2.305	–0.384

	natural electron configuration of Pt
PtF	6s(0.56) 6p(0.07) 7p(0.03) 8p(0.01) 5d(8.80) 6d(0.01)
PtF_2	6s(0.88) 6p(0.06) 7p(0.02) 8p(0.01) 5d(8.03) 6d(0.01) 5f(0.01)
PtF_3	6s(0.49) 6p(0.07) 7p(0.03) 8p(0.01) 5d(7.92) 6d(0.01) 5f(0.01)
PtF_4	6s(0.54) 6p(0.07) 7p(0.03) 8p(0.01) 5d(7.57) 6d(0.01) 5f(0.02)
PtF_5	6s(0.33) 6p(0.07) 7p(0.03) 8p(0.01) 5d(7.41) 6d(0.01) 5f(0.03)
PtF_6	6s(0.37) 6p(0.07) 7p(0.03) 8p(0.01) 5d(7.24) 6d(0.02) 5f(0.03)

Part 8 Additional scalar-relativistic DFT calculations on PtF_n (n = 1–6) and PtF_4 complexes

Table S8.1 DFT computed structure and vibrational frequencies (in cm^{-1}) for PtF_n (n = 1–5)

PtF ($X^2\Pi$, $C_{\infty v}$), B3LYP/AVTP(-PP): r(Pt–F) = 1.883 Å

^{194}Pt (int.)	^{195}Pt (int.)	^{196}Pt (int.)	^{198}Pt (int.)	Modes
569(67)	569(67)	569(67)	569(67)	ν (Pt-F), Σ^+

PtF_2 ($X^3\Sigma_g^-$, $D_{\infty h}$), B3LYP/AVTP(-PP): r(Pt–F) = 1.836 Å

^{194}Pt (int.)	^{195}Pt (int.)	^{196}Pt (int.)	^{198}Pt (int.)	Modes
720(158)	720(157)	720(157)	719(157)	Σ_u^+
662(0)	662(0)	662(0)	662(0)	Σ_g
166(4)	166(4)	166(4)	166(4)	Π_u

PtF_3 (X^2A_2, C_{2v}), B3LYP/AVTP(-PP), r(Pt–F) = 1.885 Å, r(Pt–F_2') = 1.866 Å, α(F–Pt–F′) = 92.3°

^{194}Pt (int.)	^{195}Pt (int.)	^{196}Pt (int.)	^{198}Pt (int.)	Modes
680(169)	680(169)	679(169)	679(169)	B_2
665(0)	665(0)	665(0)	665(0)	A_1
631(27)	631(27)	631(27)	631(27)	A_1
243(5)	243(5)	243(5)	243(5)	A_1
127(4)	127(4)	127(4)	127(4)	B_2
111(10)	111(10)	111(10)	111(10)	B_1

PtF_4 (X^3B_{2g}, D_{2h}), B3LYP/AVTP(-PP), r(Pt–F) = 1.854 Å, α(F–Pt–F) = 87.6°

^{194}Pt (int.)	^{195}Pt (int.)	^{196}Pt (int.)	^{198}Pt (int.)	Modes
706(146)	705(146)	705(145)	704(145)	B_{3u}
694(156)	693(156)	693(155)	693(155)	B_{2u}
679(0)	679(0)	679(0)	679(0)	A_g
272(0)	272(0)	272(0)	272(0)	B_{1g}
254(7)	254(7)	254(7)	254(7)	A_g
250(0)	250(0)	250(0)	250(0)	B_{3u}
236(10)	236(10)	236(10)	236(10)	B_{1u}
222(12)	222(12)	222(12)	222(12)	B_{2u}
164(0)	164(0)	164(0)	164(0)	A_u

PtF_5 (X^2B_2, C_{4v}), B3LYP/AVTP(-PP), r(Pt–F) = 1.860 Å, r(Pt–F_4') = 1.873 Å, α(F–Pt–F′) = 92.3°

^{194}Pt (int.)	^{195}Pt (int.)	^{196}Pt (int.)	^{198}Pt (int.)	Modes
687(5)	687(5)	687(5)	686(5)	A_1
679(151)*2	679(151)*2	679(151)*2	678(150)*2	E
662(1)	662(1)	662(1)	662(1)	A_1
619(0)	619(0)	619(0)	619(0)	B_2
267(10)	267(10)	267(10)	266(10)	A_1
259(0)	259(0)	259(0)	259(0)	B_1
230(13)*2	230(13)*2	230(13)*2	230(13)	E
204(0)	204(0)	204(0)	204(0)	B_2
102(1)*2	102(1)*2	102(1)*2	102(1)*2	E

Part 9 Scalar-relativistic CCSD(T) calculations on PtF_n (*n* = 1–5)

PtF ($X^2\Pi$, $C_{\infty v}$), CCSD(T)/AVTP(-PP), *r*(Pt–F) = 1.880 Å

^{194}Pt	^{195}Pt	^{196}Pt	^{198}Pt	Modes
578	578	578	578	ν (Pt-F), Σ^+

PtF_2 ($X^3\Sigma_g^-$, $D_{\infty h}$), CCSD(T)/AVTP(-PP), *r*(Pt–F) = 1.830Å

^{194}Pt	^{195}Pt	^{196}Pt	^{198}Pt	Modes
732	731	731	730	Σ_u^+
669	669	669	669	Σ_g
165*2	165*2	165*2	165*2	Π_u

PtF_3 (X^2A_2, C_{2v}), CCSD(T)/AVTP(-PP), *r*(Pt–F) = 1.875 Å, *r*(Pt–F$_2$′) = 1.854 Å, *α*(F–Pt–F′) = 92.1°

^{194}Pt	^{195}Pt	^{196}Pt	^{198}Pt	Modes
703	702	702	701	B_2
685	685	685	685	A_1
644	644	644	644	A_1
251	251	251	251	A_1
144	144	144	143	B_2
128	128	128	128	B_1

PtF_4 (X^3B_{2g}, D_{2h}), CCSD(T)/AVTP(-PP), *r*(Pt–F) = 1.838 Å, *α*(F–Pt–F) = 87.4°

^{194}Pt	^{195}Pt	^{196}Pt	^{198}Pt	Modes
731.92	731.60	731.29	730.66	A_1
718.25	717.95	717.66	717.08	B_2
705.02	705.02	705.02	705.02	A_1
273.93	273.93	273.93	273.93	A_1
264.93	264.87	264.80	264.68	A_1
235.95	235.78	235.61	235.28	B_1
215.15	215.09	215.04	214.93	B_2
169.18	169.18	169.18	169.18	A_2
63.59	63.59	63.59	63.59	B_2

PtF_5 (X^2B_2, C_{2v}), CCSD(T)/AVTP(-PP), r(Pt–F) = 1.854 Å, r(Pt–F$_4'$) = 1.856 Å, α(F–Pt–F) = 91.9°

^{194}Pt	^{195}Pt	^{196}Pt	^{198}Pt	Modes
706*2	706*2	705*2	705*2	E
689	689	689	689	A_1
675	675	675	675	A_1
634	634	634	634	B_2
276	276	276	275	A_1
266	266	266	266	B_1
229*2	229*2	229*2	229*2	E
214	214	214	214	B_2
124*2	124*2	124*2	124*2	E
